# Search and processing of Holliday junctions within long DNA by junction-resolving enzymes

**DOI:** 10.1038/s41467-022-33503-6

**Published:** 2022-10-07

**Authors:** Artur P. Kaczmarczyk, Anne-Cécile Déclais, Matthew D. Newton, Simon J. Boulton, David M. J. Lilley, David S. Rueda

**Affiliations:** 1 Department of Infectious Disease, Faculty of Medicine, Imperial College London, London, W12 0NN UK; 2 Single Molecule Imaging Group, MRC-London Institute of Medical Sciences, London, W12 0NN UK; 3 School of Life Sciences, University of Dundee, Dundee, DD1 5EH UK; 4 DSB Repair Metabolism Laboratory, The Francis Crick Institute, London, NW1 1AT UK

**Keywords:** Single-molecule biophysics, DNA, Enzyme mechanisms

## Abstract

Resolution of Holliday junctions is a critical intermediate step of homologous recombination in which junctions are processed by junction-resolving endonucleases. Although binding and cleavage are well understood, the question remains how the enzymes locate their substrate within long duplex DNA. Here we track fluorescent dimers of endonuclease I on DNA, presenting the complete single-molecule reaction trajectory for a junction-resolving enzyme finding and cleaving a Holliday junction. We show that the enzyme binds remotely to dsDNA and then undergoes 1D diffusion. Upon encountering a four-way junction, a catalytically-impaired mutant remains bound at that point. An active enzyme, however, cleaves the junction after a few seconds. Quantitative analysis provides a comprehensive description of the facilitated diffusion mechanism. We show that the eukaryotic junction-resolving enzyme GEN1 also undergoes facilitated diffusion on dsDNA until it becomes located at a junction, so that the general resolution trajectory is probably applicable to many junction resolving enzymes.

## Abstract (web summary)

Locating a four-way junction in a high background of genomic DNA is likely to be the rate-limiting step of the resolution process. This study captures the entire reaction trajectory of a nuclease targeting and resolving a DNA junction at single-molecule level.

## Introduction

**Fig. 1:**
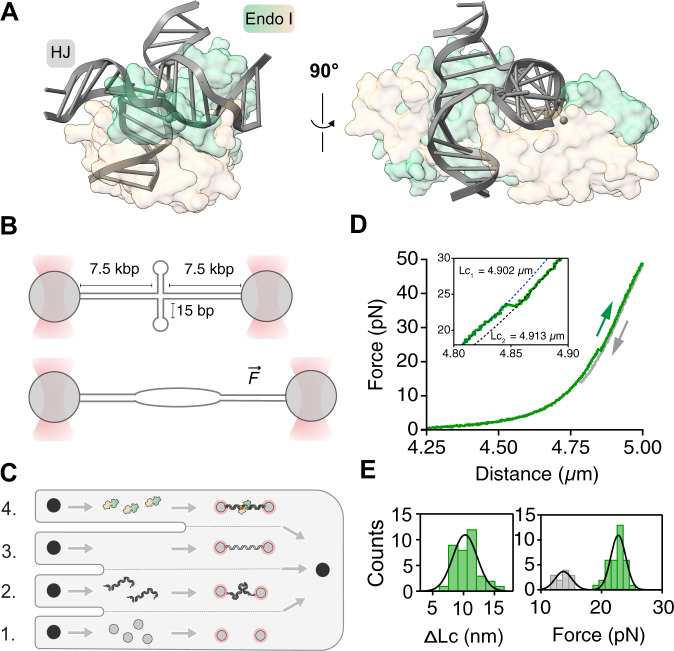
A four-way junction located at the centre of a 15 kbp dsDNA is stable below 20 pN. **A** Structure of the phage T7 endonuclease I homodimer (pale yellow and green) bound to a four-way junction (grey). **B** Schematic of a dumbbell assembly. Biotinylated 15 kbp DNA with a four-way junction at its centre is tethered between optically trapped, streptavidin-coated polystyrene beads. Increasing the distance between the two beads generates a tensile force on the DNA leading to unfolding of the four-way junction. **C** Schematic of the microfluidic device used to trap, reconstitute and image the endonuclease bound to the DNA: (1) beads channel; (2) DNA channel; (3) buffer channel; (4) protein channel. **D** Force-extension curve (F-E) of the tethered DNA stretched from 0 to 50 pN (green) and subsequently relaxed to 0 pN (grey). The cycle was performed in the buffer channel. Inset: zoom on the discontinuity in the F-E curve which represents the unfolding of the four-way junction. Blue and black dotted lines are the extensible Worm-Like-Chain model fitted from the F-E fragments before and after the unfolding transition, respectively. **E** *Left panel*: Distribution of the contour length change (ΔLc) measured on individual molecules. The average change in contour length, 9.5 ± 0.3 nm (mean + S.E.M., *n* = 36), was calculated by fitting the distribution with a Gaussian function. *Right panel*: Distribution of the measured forces associated with unfolding and refolding the junction (green and grey histogram, respectively). The four-way junction is stable below 23 ± 1 pN (mean ± S.E.M., *n* = 35). The secondary structure becomes refolded when the DNA is relaxed below 14 ± 1 pN of force (mean ± S.E.M., *n* = 13). Both averages were calculated by fitting the histograms with a Gaussian function. Source data are provided as a Source Data file.

Homologous genetic recombination is critically important to genome maintenance, for the precise repair of double-strand breaks, and the processing of stalled replication forks, as well as in facilitating chromosome segregation in meiosis^1^. The central intermediate in these processes is the four-way Holliday junction in which four helices are connected via the covalent continuity of the DNA strands^2,3^. These junctions can be created by several pathways, but ultimately, they must be processed into two separated duplex species. Holliday junctions can be removed by dissolution^4^, requiring the action of a helicase and a topoisomerase, or resolution by a junction-selective nuclease^5^. Junction-resolving enzymes are widespread, having been isolated from bacteria and their phages, mitochondria, archaea and eukaryotes^6^. They are dimeric nucleases that selectively bind with nanomolar affinity to four-way DNA junctions and then introduce two symmetrically-paired hydrolytic cleavages (Fig. 1A). We have found that, in general, these two cleavage events are sequential, but the second cleavage is accelerated 10–100 fold faster than the first, so that both cleavages are made within the lifetime of the DNA-protein complex ensuring productive resolution^7,8^.

The first phase of the resolution process in which the resolving enzyme locates and binds to a junction is poorly understood. This requires that the enzyme finds a four-way junction within a relatively large length of double-stranded DNA. A similar problem is faced by a variety of sequence-specific DNA binding proteins such as transcription factors, restriction enzymes and methylases that must locate their targets within long dsDNA molecules. Two search processes are possible: First, the protein could diffuse in three-dimensions within the volume of the cell or nucleus and rely on chance encounters to meet the target and then bind. Second, the protein might bind to double-stranded DNA at a random location and then undergo facilitated one-dimensional diffusion until it encounters its target. The latter should accelerate the location of a junction by lowering the dimensionality of the search process.

Facilitated diffusion was first proposed^9–11^ to explain the observation that the Lac repressor apparently locates its target faster than could be explained by 3D diffusion^12^, and the theory was extended to DNA binding proteins more generally^13^. Facilitated diffusion includes sliding, where the protein remains in contact with the DNA while undergoing bi-directional translocation along the contour of the DNA, and hopping, where the protein transiently dissociates before rebinding a small distance away, i.e., it undergoes microscopic hops. In addition, for proteins that have two potential DNA binding domains, intersegmental transfer may be possible if the DNA can form a loop. While the original deduction that DNA binding proteins undergo facilitated diffusion was based on detailed kinetic analysis of binding experiments in bulk solution, it has become possible to study this more directly by single-molecule tracking experiments. For the most part, the movement of fluorescently-labelled proteins has been studied on DNA molecules stretched by flow, and 1D diffusion of proteins along the contour of dsDNA has been observed for human oxoguanine glycosylase^14^, LacI repressor^15^, Rad51^16^ and p53^17^. Diffusion along DNA of the human mitochondrial transcription factor A^18^, the origin replication complex^19^ and Cas12a^20^ have been observed using DNA held under tension with two optical traps. In general, a wide range of 1D diffusion rates have been observed; for example, reported diffusion constants range from D ~ 10^3^ bp^2^·s^−1^ for LacI^15^ and Rad51^16^ to ~ 10^6^ bp^2^·s^−1^ for p53^17^ and hOgg1^14^.

It is not known if facilitated diffusion is possible for a structure-selective DNA binding protein, but in principle a junction-resolving enzyme might locate its target four-way junction in this manner. We know that resolving enzymes have some affinity for dsDNA, typically two to three orders of magnitude lower than that for a four-way junction. In general, junction-resolving enzymes alter the structure of the DNA in a major way, both in terms of the disposition of the helices and the local base pairing^21–25^, and the binding interfaces are generally highly basic^26–28^, consistent with an electrostatic interaction with the DNA. It is conceivable that they could interact with dsDNA in a way that permits a sliding motion, allowing movement along the contour of the dsDNA until a junction is encountered.

We, therefore, set out to explore this possibility with the junction-resolving enzyme bacteriophage T7 endonuclease I. This enzyme was fluorescently labelled, and its interaction with a long dsDNA molecule containing a central four-way junction studied using a combination of optical trapping and confocal microscopy in a microfluidic cell^29^. We directly observe random bidirectional diffusion of the protein along the dsDNA until it encounters the junction, whereupon it remains stationary at that point. When active endonuclease I is employed then after a period we observe a cleavage event. Thus, in a single experiment, we can observe a complete trajectory, from initial remote binding to dsDNA, facilitated diffusion to locate the junction and ultimately cleavage of the junction in the resolution reaction.

## Results

### Characterisation of a 15 kbp dsDNA molecule with a central four-way junction

To study the location and association of a junction-resolving enzyme with a four-way (Holliday) junction, we first constructed a 15 kbp DNA molecule with a centrally-positioned double 15 bp hairpin junction with 5 nt closing loops (Fig. 1B, Supplementary Fig. 1, and Materials and Methods). To characterise the mechanical stability of the junction-containing DNA, we assembled a dumbbell by immobilising the DNA between two optically trapped beads using a microfluidics cell (Fig. 1B, C)^20,30,31^. This dumbbell enables us to apply mechanical stretching force along the length of the DNA. By measuring the applied force as a function of bead-to-bead distance, we generated a force-extension curve (Fig. 1D, green), that was fitted to the extensible Worm-Like-Chain model^32^ (eWLC) to yield the DNA contour length of 4.902 ± 0.006 µm (Fig. 1D, inset). At ~23 pN, the curve deviated from the initial eWLC model indicating an abrupt increase in contour length of 9.5 ± 0.3 nm on average (Fig. 1D, inset and Fig. 1E, left, *n* = 36 DNA tethers), in good agreement with the length increase expected for unzipping of the two 15 bp hairpins.

Once the junction has become mechanically unfolded, it will undergo refolding upon lowering the force (Fig. 1D, grey). The junction does not re-fold until the force is lower than 14 ± 1 pN (Fig. 1E, grey, *n* = 13, mean ± S.E.M.), showing a hysteresis in the junction’s unfolding-refolding cycle. Since the stretching curve does not overlap with the relaxation curve, the system is not in equilibrium under our conditions. The pulling rate is too fast compared to the slowest relaxation rate in the molecule, and the unfolding transition is then considered as irreversible. In this situation, the Gibbs free energy change is no longer equal to the work done on the molecule^33^. However, the free energy of the junction can be estimated, as described^34^, where the area under the unfolding event is measured to assess the mechanical stability of a four-way junction. This analysis on the force-distance data shown in Fig. 1D yields a ΔG_area_ = 37 kcal/mol, which is smaller than the 53 ± 13 kcal/mol value reported a for a longer (24 bp stem) double-hairpin construct^34^. Together, these data confirm the presence of a four-way junction in our DNA construct, that is mechanically stable below a stretching force of 23 ± 1 pN (Fig. 1E, right, *n* = 35). In subsequent experiments, we maintain a constant force of 5 pN to keep the DNA gently stretched in a linear form, but without disrupting the structure of the junction.

### Endonuclease I searches for four-way junctions by 1D diffusion before cleavage

**Fig. 2:**
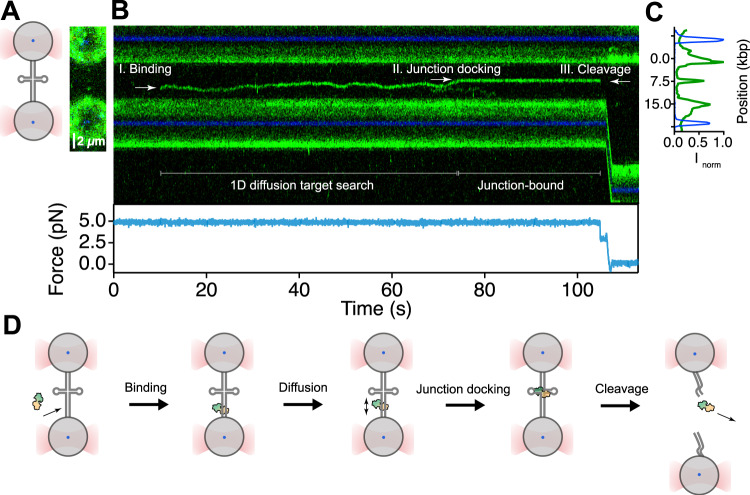
A complete reaction trajectory in which endonuclease I diffuses on the dsDNA until it encounters and cleaves a four-way junction. **A** *Left panel*: Schematic of the dumbbell assembly. The DNA with a four-way junction at its centre is tethered between optically trapped beads. *Right panel:* A confocal image of the dumbbell with endonuclease I (green dots) bound to the DNA (unlabelled). **B** A stack of frames (kymograph) recorded by continuous confocal scanning along the DNA axis. Corresponding force exerted on the DNA, plotted below, is kept constant by a feedback loop mechanism and decreases to 0 pN upon DNA cleavage. The intermediate force level at 3 pN is likely an artifact from the force feedback loop mechanism, and is not observed in other kymographs (Supplementary Fig. 5). The kymograph shows a complete trajectory of the endonuclease I performing random walk on the stretched DNA followed by its docking on the four-way junction leading to the DNA cleavage. The green areas with the blue lines reveal the positions of the optically trapped beads. **C** Cumulative fluorescence intensity profile of the kymograph (90–110 s window). The central peak corresponds with the junction-bound endonuclease I. The position on the DNA (in kbp) was interpolated from the pixel position using the bead centres (blue peaks) as a reference. **D** A cartoon representation of the complete reaction trajectory of endonuclease I target search and cleavage activity described above.

After characterising the mechanical stability of the four-way junction in our 15 kbp DNA construct, we investigated the behaviour of single T7 endonuclease I enzymes on force-stretched DNA using fluorescence confocal microscopy^20,30^. Endonuclease I was Cy3-labelled at a single cysteine on each endonuclease I monomer (Supplementary Fig. 2, Materials and Methods). Labelling did not significantly affect the enzyme’s activity (Supplementary Fig. 3). The assembled dumbbell DNA with its central junction was moved into a microfluidic channel containing T7 endonuclease I and clamped at 5 pN force, where enzyme binding could be readily detected as fluorescent spots (Fig. 2A). To investigate the dynamics of the DNA-bound complex, we measured the fluorescence intensity along the DNA axis as a function of time to generate a kymograph (Fig. 2B), which shows the movement of endonuclease I on the DNA. The kymograph shows an endonuclease I dimer binding the double-stranded DNA as a green signal appearing after 10 s (Fig. 2B, D). Two-step photobleaching analysis confirmed that the diffusing species is a dimer of endonuclease I, as expected^35^ (Supplementary Fig. 4A). Initially, the dimer binds to duplex DNA far from the four-way junction. Subsequently, the fluorescent protein moves randomly and bidirectionally on the stretched DNA, indicating 1D diffusion. The protein remains in motion until making a chance encounter with the centrally located junction (Fig. 2B, D). The exact position of the docking site was confirmed by plotting the cumulative fluorescence intensity as a function of its position on the DNA (Fig. 2C). Upon encountering the junction, the endonuclease remains stably bound for almost 30 s before cleaving the four-way junction (Fig. 2B, D). Under our force-clamp conditions, DNA cleavage results in a reduction in the measured force (Fig. 2B, bottom). Simultaneously, the fluorescent signal of the protein disappears from the kymograph, and subsequently separation of the beads occurs because the DNA tether has been cleaved (Supplementary Fig. 5). These results demonstrate that we can capture an entire reaction trajectory of the enzyme, starting from initial binding at a remote random DNA site, followed by 1D diffusion along the DNA, junction recognition and its subsequent cleavage.

### Facilitated diffusion of Endonuclease I on dsDNA

**Fig. 3:**
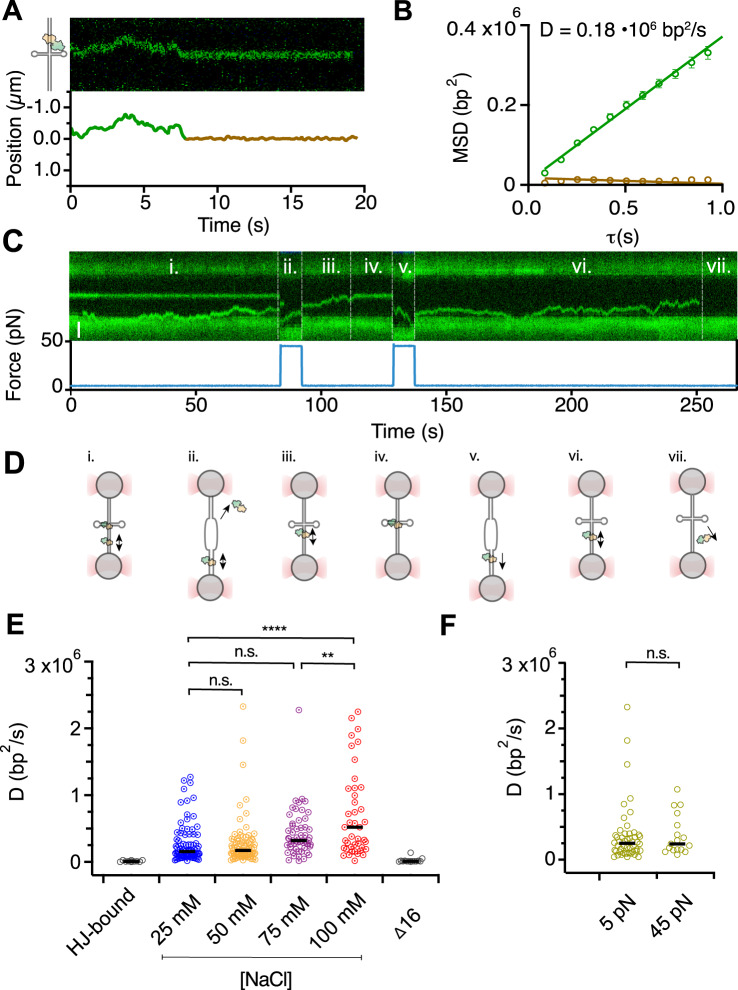
Endonuclease I docking is force dependent and its diffusion is salt-dependent. **A** *Top panel:* A kymograph showing the endonuclease I (catalytically impaired mutant D55A) that diffuses on dsDNA and docks on a four-way junction. *Bottom panel:* The diffusing and static segments of the trajectory (green and brown, respectively) resolved by single-particle Gaussian tracking. **B** Mean-square displacement (MSD) plot of the trajectories shown in panel **A**. Linear fit of the MSD dependency over intervals 0.2 s ≤ τ ≤ 1 s yields the diffusion coefficient of the diffusing endonuclease I (D = 0.021 ± 0.001 μm^2^s^−1^ = 0.18·10^6^ bp^2^s^−1^, green). The MSD of the static, junction-bound complex does not increase (D = (0.01 ± 0.01)·10^6^ bp^2^s^−1^, brown). Error was calculated as standard error of the linear fit (S.E). **C** A kymograph recorded at 4 and 45 pN (force measurement plotted below). At low tension the endonuclease I is either stably bound to the centrally located four-way junction or diffuses along the duplex DNA. At high tension, the junction-bound complex dissociates or diffuses away due to the unfolding of junction. **D** A cartoon representation of the optically trapped DNA with the endonuclease I (D55A) during the course of the experiment presented in panel C. **E** Distribution of the diffusion coefficients of endonuclease I at different ionic strengths in comparison with static molecules. Black circles: Endonuclease I complexes that are bound to the four-way junction do not diffuse and the calculated apparent diffusion rates reflect the detection limit of the Gaussian tracking (D_HJ_ = (0.01 ± 0.01) · 10^6^ bp^2^s^−1^, median ± S.E.M., *n* = 11); blue circles: diffusion rates of individual endonuclease I molecules in the presence of 25 mM NaCl (D_25_ = (0.15 ± 0.05)·10^6^ bp^2^s^−1^, median ± S.E.M., *n* = 85); yellow circles: diffusion rates of individual endonuclease I molecules in the presence of 50 mM NaCl (D_50_ = (0.17 ± 0.04)·10^6^ bp^2^s^−1^, median ± S.E.M., *n* = 79); violet: diffusion rates of endonuclease I molecules in the presence of 75 mM NaCl (D_75_ = (0.32 ± 0.05)·10^6^ bp^2^s^−1^, median ± S.E.M, n = 54); red circles: diffusion rates of endonuclease I molecules in the presence of 100 mM NaCl (D_100_ = (0.52 ± 0.11) · 10^6^ bp^2^s^−1^, median ± S.E.M., *n* = 43); grey circles: diffusion rates of Δ16-endonuclease I mutant in 50 mM NaCl buffer (D_Δ16_ = (0.01 ± 0.01)·10^6^ bp^2^s^−1^, median ± SD, *n* = 15). The average diffusion rate at the highest salt concentration (100 mM) is significantly different from the diffusion rate at low salt (25 mM) and medium salt (75 mM) concentration; *p* = 0.0001 and *p* = 0.0047, respectively (two-sided *t*-test, box plot flagged with 4-stars (****) indicates a *p* < 0.0001 and 2 stars (**) indicates a *p* < 0.01, “n.s.” stands for “not significantly different”). **F** Distribution of the diffusion coefficients of endonuclease I on λ-DNA under 5 pN and 45 pN tensile force. The rate at low force (D_5pN_ = (0.25 ± 0.01)·10^6^ bp^2^s^−1^ (median + S.E.M)) is the same as the rate at high force (D_45pN_ = (0.24 ± 0.07) ·10^6^ bp^2^s^−1^ (median + S.E.M.)). Source data are provided as a Source Data file.

As shown above, endonuclease I initially binds to duplex DNA remotely from the junction, and then moves by bidirectional 1D diffusion along the dsDNA. To characterise the diffusion dynamics of endonuclease I on the dsDNA, we extracted the trajectory of fluorescent molecules using a custom-made single-particle tracking algorithm (Fig. 3A). We calculated the mean-square displacement (MSD) of the translocating enzyme before and after binding the junction (Fig. 3B). Prior to junction-docking, the MSD increases linearly with the time interval (Fig. 3B, green), indicating that endonuclease I translocates on the dsDNA by 1D diffusion. The diffusion coefficient is proportional to the slope of the MSD curve, from which we calculate D = 0.18 · 10^6^ bp^2^·s^−1^ (Materials and Methods). Conversely, the MSD of static junction-bound complexes does not increase (Fig. 3B, brown).

To prove that static molecules are located directly on the junction, we disrupted the junction by briefly increasing the force from 5 to 45 pN (Fig. 3C), reaching a level of stretching tension that is significantly above its mechanical stability (Fig. 1D, E). While at low force, endonuclease I can readily locate the central junction (Fig. 3C, D); increasing the force after time (t) ~ 80 s results in a loss of fluorescent signal indicating endonuclease I dissociation from the junction. Decreasing the force back to 5 pN (at t ~ 90 s) allows a second diffusing complex to find and bind to the re-folded junction (at t ~ 115 s). Upon increasing the force a second time to 45 pN (at t ~ 130 s), endonuclease I again undocks and diffuses away from the junction until the protein dissociates or the dyes become photobleached (at t ~ 250 s) (Fig. 3C, D).

To investigate the diffusion behaviour of the enzyme on dsDNA in the absence of a junction, we changed to a long (48.5 kbp) DNA substrate (bacteriophage λ-DNA). We tracked endonuclease I diffusion for up to ~5 min on this substrate, limited only by Cy3-photobleaching (Supplementary Fig. 4A). On average, the enzyme spent 55 ± 9 s on dsDNA surveying about 0.54 ± 0.03 kbp of sequence per second (Supplementary Fig. 4B, C). To characterise further the mode of endonuclease I diffusion (sliding, hopping or potentially a hybrid of the two) on the DNA, we measured the diffusion coefficient in the presence of different NaCl concentrations. Owing to DNA screening by cations, hopping is expected to depend on salt concentration, whereas sliding is not^36^. We probed diffusion of endonuclease I in the presence of 25 to 100 mM NaCl. The diffusion coefficient of individual endonuclease I molecules extends over a broad range of values from ~0.2 · 10^6^ bp^2^·s^−^^1^ to 3 · 10^6^ bp^2^·s^−1^ (Fig. 3E, *n* = 261). At low and intermediate salt concentrations (25 and 50 mM), the respective median diffusion rates are (0.15 ± 0.05) · 10^6^ bp^2^·s^−1^ (*n* = 85, median ± S.E.M.) and (0.17 ± 0.04) · 10^6^ bp^2^·s^−1^ (*n* = 79). But at higher ionic strength (100 mM), the median diffusion rate increased significantly to (0.5 ± 0.1) · 10^6^ bp^2^·s^−1^ (*n* = 79), indicating that, at higher salt concentrations, endonuclease I spends more time in solution as unbound and mobile, possibly consistent with a hopping mechanism^17^. In some kymographs (*n* = 10), we observed long jumps of 1–3 µm (~3–9 kbp) in less than 0.1 s which display the enzyme hopping, or “slipping”^37^, large distances on dsDNA with diffusion rates exceeding temporal resolution of the measurement (Supplementary Fig. 6). At salt concentrations higher than 100 mM, residence times of the proteins on the DNA became too short to permit the determination of diffusion coefficients. In contrast, increasing the force from 5 to 40 pN at 50 mM salt did not alter the observed diffusion coefficients significantly (D_45pN_ = (0.24 ± 0.07) · 10^6^ bp^2^·s^−1^, Fig. 3F, *n* = 18, see also Supplementary Fig. 7). Altogether, these data show that endonuclease I diffusion is salt-dependent but not force-dependent.

### Cleavage kinetics of endonuclease I on a junction

**Fig. 4:**
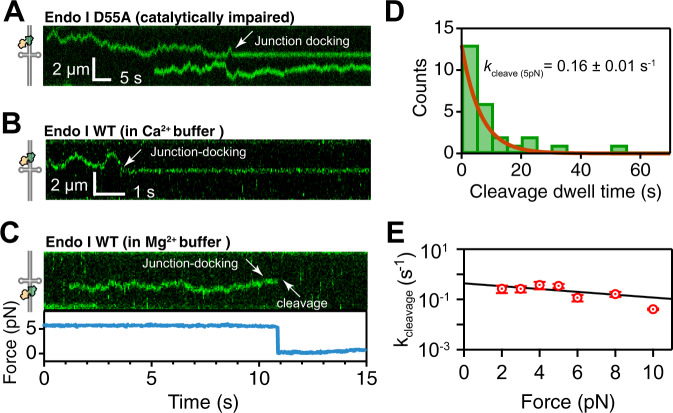
Observation of catalytically-active endonuclease I resolving a four-way junction. **A** A kymograph showing the catalytically impaired endonuclease I D55A mutant that diffuses on dsDNA until it encounters the centrally-located four-way junction. Once docked on its target, the protein remains bound for long periods of time. **B** A kymograph showing the wild type endonuclease I in conditions that supress its enzymatic activity. The protein diffuses on dsDNA, remains docked on the four-way junction without cleaving the DNA. **C** A kymograph showing the wild type endonuclease I in a buffer with Mg^2+^ cations. The corresponding force measurement is plotted below. 2 s after docking on the four-way junction, the fluorescence signal disappears and the force drops to 0 pN, indicating that the DNA was cleaved. **D** Distribution of the endonuclease I dwell times on the junction prior to the observation of cleavage (*n* = 26, *F* = 5 pN). Exponential fit of the histogram (red) reveals the cleavage rate *k*_cleave_ = 0.16 ± 0.01 s^−1^. **E** Dependence of *k*_cleave_ on the applied force (red). Linear fit with the Bell-Evans equation (black) yields force-independent cleavage rate constant *k*_cleave_(0) = 0.4 ± 0.1 s^−1^, and the distance to the transition state (Δx^‡^ = 3 ± 2 Å). Source data are provided as a Source Data file.

The entire reaction trajectory of the enzyme (Fig. 2) shows that endonuclease I remains statically bound at the junction between docking and cleavage. Cleavage was never observed with a catalytically inactive mutant (endonuclease I D55A, Fig. 4A), in the absence of Mg^2+^ ions or in the presence of Ca^2+^ ions, as expected (Fig. 4B). The residence time docked at the junction varies stochastically from molecule to molecule (e.g., compare Figs. 2B and 4C). To estimate the cleavage rate constant, we measured the distribution of dwell times at the junction from initial docking to cleavage at 5 pN (Fig. 4D). Although junction resolution occurs in two sequential cleavage steps^7,8^, we observed a single exponential dwell time distribution, consistent with the first cleavage being rate-limiting and the second cleavage occurring much faster than the first^35^. An exponential fit of the dwell time distribution yields a cleavage rate constant *k*_cleave_ = 0.16 ± 0.01 s^−1^ (Fig. 4D, *n* = 26). Further, we measured the dependence of *k*_cleave_ with force in the 2–10 pN range. The resulting rate constants (Fig. 4E) show that *k*_cleave_ decreases slightly with increasing force, indicating that tension on the DNA hinders cleavage slightly. A fit to the Bell-Evans equation^20,38^ yields the zero-force cleavage rate constant *k*_cleave_(0) = 0.4 ± 0.1 s^−1^, in good agreement with bulk measurements^7,8^, and the length of activation (Δx^‡^ = 3 ± 2 Å), which indicates a very small conformational change to reach the transition state of cleavage from the docked state.

### The N-terminal peptide of endonuclease I is important for facilitated diffusion

**Fig. 5:**
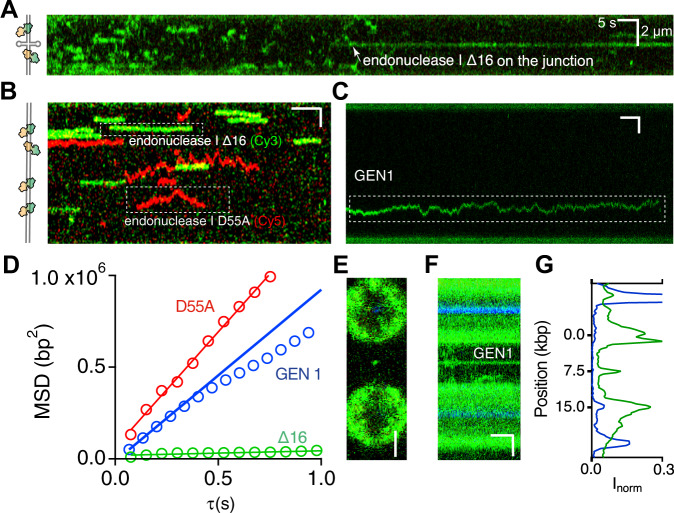
The role of the N-terminal peptide of endonuclease I, and generalisation of the 1D diffusion on dsDNA to a cellular resolving enzyme. **A** A kymograph showing the truncated endonuclease I (Endo I-Δ16) interacting with the DNA that has a centrally located four-way junction. Endonuclease mutants present in the buffer bind transiently to the duplex DNA. The molecule that docks on the four-way junction at later stage of imaging (after ~ 75 s) remains bound to it for over a minute. **B** A kymograph showing simultaneous binding of endonuclease I Δ16 (Cy3-labelled, green) and endonuclease I D55A (Cy5-labelled, red) on λ-DNA. **C** A kymograph showing C. thermophilum GEN1 undergoing 1D diffusion on λ-DNA. **D** Mean-square displacement (MSD) plot of the selected trajectories shown in panel **B** and **C**. Linear fit to the MSD dependency yields the diffusion coefficient of diffusing endonuclease I Δ16 (D_Δ16_ = 0.01·10^6^ bp^2^s^−1^, green), endonuclease I D55A (D_D55A_ = 0.6·10^6^ bp^2^s^−1^, red), and GEN1 (D_GEN1_ = 0.5·10^6^ bp^2^s^−1^ blue). **E** A confocal image of the dumbbell with GEN1 (green spot at the centre) bound to the DNA (unlabelled). Scale bar corresponds to 2 μm. **F** A kymograph showing GEN1 docked on a centrally-positioned four-way junction. **G** Cumulative fluorescence intensity profile of the kymograph in panel **F**. Central peak corresponds with the junction-bound GEN1. The position on the DNA (in kbp) was interpolated from the pixel position using the bead centres (blue peaks) as a reference.

Our previous studies have shown that deletion of the predominantly basic 16 amino acids from the N-terminus of T7 endonuclease I (endonuclease I ∆16) led to tighter binding but slower cleavage of four-way DNA junctions^39^. Although the N-terminal peptide was not visible in crystal structures with or without a DNA junction^26,40^, it is clearly important for binding to the four-way junction^41^. To investigate the function of this peptide, we incubated endonuclease I lacking the first 16 N-terminal amino acids with the junction-containing DNA, and measured its diffusive behaviour in the presence of Ca^2+^ to prevent cleavage. The resulting kymographs revealed a markedly different behaviour from the full-length enzyme complex (compare Figs. 2 and 5A). In the majority of observed binding events, the residence time of endonuclease I Δ16 bound to the DNA decreases at least 10-fold and its diffusion on the dsDNA decreases considerably (D_∆16_ = (0.01 ± 0.01) · 10^6^ bp^2^·s^−1^, Figs. 3E and 5D). Interestingly, when endonuclease I Δ16 successfully locates the junction, it can remain stably bound for a long time (several minutes) (Supplementary Fig. 8). However, the junction is located through a random collision rather than by facilitated diffusion as for the full-length complex.

To highlight further the differences in diffusion between endonuclease I Δ16 and the full-length enzyme, we performed a two-colour experiment with Cy3-labelled endonuclease I Δ16 (green) and enzymatically-inactive Cy5-labelled D55A endonuclease I (red) on λ-DNA (Fig. 5B). The resulting kymograph clearly shows that only the catalytically inactive mutant diffuses along the DNA, whereas endonuclease I Δ16 remains almost static at its initial binding site. These results confirm our findings and indicate that the N-terminal peptide is involved in promoting long facilitated diffusion on dsDNA to locate its substrate rapidly. A possible mechanism to achieve this could be by holding the endonuclease I dimer onto the dsDNA as discussed below. The weaker interaction of endonuclease I ∆16 with dsDNA would thereby favour dissociation over 1D diffusion. Consequently, the likelihood of finding a junction in long (kbp) dsDNA would decrease significantly, thus slowing down the overall cleavage rate observed in bulk studies.

### Eukaryotic junction-resolving enzyme GEN1 undergoes facilitated diffusion along dsDNA

T7 endonuclease I is a phage-encoded nuclease whose function is to resolve junctions remaining in replicated phage DNA. By contrast, the cellular junction-resolving enzymes from bacteria to humans function to resolve Holliday junctions generated during DNA repair, replication and recombination^5^. For example, GEN1 is a dimeric nuclease specific for four-way DNA junctions formed in eukaryotic cells^42,43^, and we have previously determined the crystal structure of this enzyme bound to its product of resolution^28^. We, therefore, asked whether GEN1 can also undergo facilitated diffusion along duplex DNA and bind to a Holliday junction within it. To this end, we prepared C. thermophilum GEN1 fluorescently-labelled at a C-terminal ybbR tag, and studied its interaction with the same DNA used above, using the confocal imaging apparatus.

Similarly to endonuclease I, GEN1 also diffuses randomly and bidirectionally on duplex DNA (Fig. 5C). We performed single-particle tracking of the mobile GEN1 molecule and obtained the diffusion coefficient D_GEN1_ = 0.5 · 10^6^ bp^2^·s^−1^ (Fig. 5D). The enzyme docks on a four-way junction (Fig. 5E–G) and remains catalytically active in Mg^2+^ containing buffer (Supplementary Fig. 9). Therefore, target search by facilitated diffusion appears to be a widely used mechanism of resolvases. Altogether, our findings highlight the importance of facilitated diffusion during DNA repair in general, and recombination, in particular.

## Discussion

Unresolved Holliday junctions can link chromosomes together, potentially resulting in a catastrophic failure to undergo cell division. For this reason, cells have evolved multiple ways to resolve DNA junctions. Junction-resolving enzymes are dimeric nucleases that specifically recognise four-way DNA junction structures. However, Holliday junctions occur relatively rarely within long double-stranded genomes. While much is known about the recognition of the junction and the manner of the cleavages, it has previously been completely unknown how resolving enzymes locate their target junctions.

**Fig. 6:**
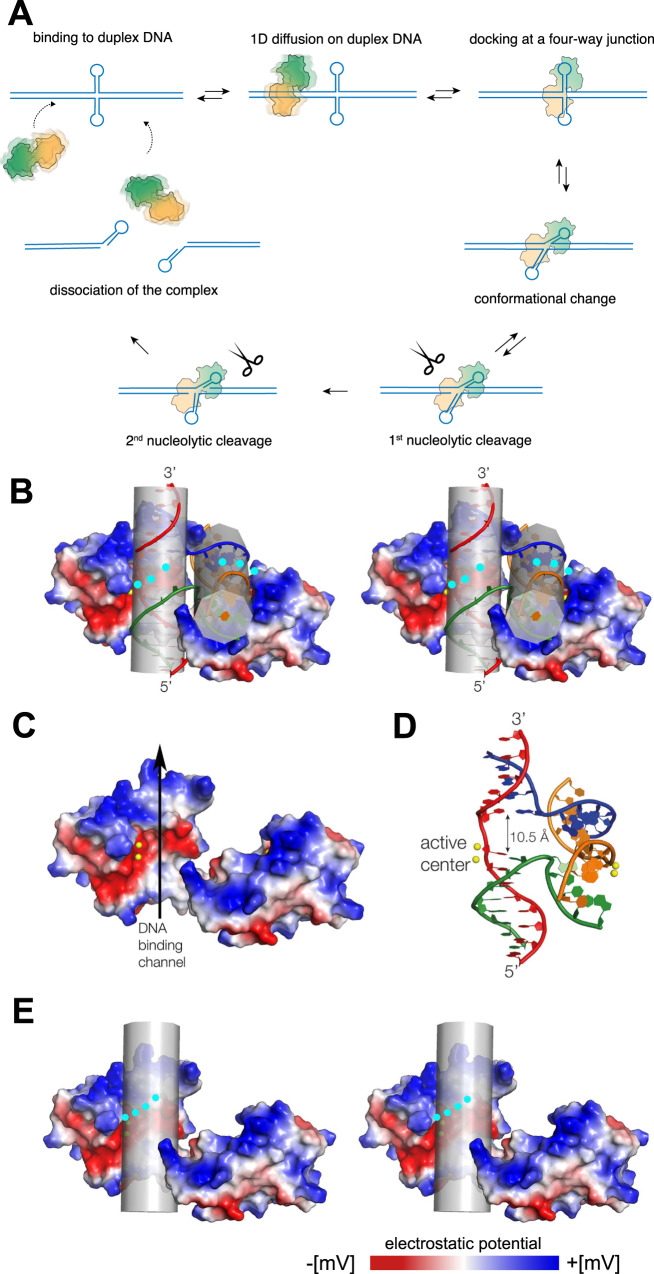
A model for the diffusion of an endonuclease I dimer on duplex DNA based on the crystal structure of the enzyme bound to a four-way junction (PDB 2PFJ, Hadden et al. 2007) and single-molecule tracking analysis. **A** A cartoon illustrating a complete reaction trajectory that leads to the resolution of a four-way junction. **B** Parallel-eye stereoscopic image of the structure of endonuclease I bound to a four-way DNA junction (PDB 2PFJ). The surface of the protein has been coloured by electrostatic potential from blue (electropositive) to red (electronegative). The position of attachment of the N-terminal 16 amino acids (not observed in the crystal) is indicated by the cyan circles; the peptides most probably interact with the DNA groove. The arms of the DNA junction form two coaxially-stacked pairs; in this view the left pair are vertical and the right pair approximately horizontal. Each pair of arms falls into a channel in the protein. **C** The structure of the endonuclease I dimer with the DNA removed to show the DNA-binding channel (shown by the arrow). As a working model we propose that duplex DNA would fit into this 34 Å-long channel, allowing translocation of the protein along the DNA. Note the strongly electropositive band at the top end of the channel in this view that forms a clamp on the DNA. The central electronegative band in part comprises the active site of the enzyme, with the two catalytic divalent metal ions show as yellow spheres. These are coordinated by three acidic amino acid side chains. **D** The structure of the DNA junction in the complex. Note that the base pairs flanking the point of strand exchange are separated, with a spacing of 10.5 Å. It is likely that this distortion of the DNA is required for accommodation into the active centre of the enzyme, so that undistorted duplex DNA is not cleaved. **E** Parallel-eye stereoscopic image of the structure of endonuclease I with a cylinder representing a bound duplex DNA molecule. The probable position of the N-terminal basic peptide is shown by the cyan circles. These most likely complete the encirclement of the duplex DNA, essentially preventing the enzyme from dissociating away from the dsDNA and allowing the protein to diffuse significant distances along the DNA.

Here, we have used optical tweezers with fluorescence detection to show that a junction-resolving enzyme from bacteriophage T7 can locate four-way junctions by first binding remotely to duplex DNA, followed by random, bidirectional one-dimensional diffusion along the DNA until it encounters a four-way junction (Fig. 6A). At this point endonuclease I remains docked at the junction. This behaviour is probably general for both viral and cellular junction-resolving enzymes because we have found that the eukaryotic enzyme GEN1 also undergoes one-dimensional diffusion until it meets a four-way junction at which point it also remains fixed to the junction. More generally, a number of other enzymes that act upon dsDNA have also been shown to undergo facilitated 1D diffusion, including p53^17^, the Lac I repressor^15^, the ORC complex^19^ and the human DNA glycosylase hOgg1^14^. One-dimensional diffusion lowers the dimensionality of the search process, thereby increasing its efficiency compared to 3D diffusion in free solution.

We have also shown that T7 endonuclease I diffuses along dsDNA in its dimeric form. This was expected because the enzyme forms a domain-exchanged dimer that is the most tightly associated amongst the known junction-resolving enzymes^35^. Once bound to the dsDNA, the protein can sample DNA lengths comparable with T7 phage genome (~ 40 kbp) in one pass with no dissociation. In free solution, binding to short DNA duplexes is orders of magnitude weaker than to a junction of the same sequence^35^. In that case, it is likely that the enzyme simply slides off the end of the duplex, which is clearly not possible when the junction is contained within a very long DNA duplex. The movement of endonuclease I along dsDNA occurs bidirectionally, with frequent changes of direction. Under our standard conditions, the diffusion coefficient is 0.18 · 10^6^ bp^2^ s^−1^. In general, the diffusion rates of other proteins observed to move along dsDNA have been measured in the range 10^3^–10^6^ bp^2^ s^−1^ ^14,15,17,19^ placing endonuclease I towards the middle of the range. The observed rate of diffusion exhibits some dependence on the prevailing salt concentration, indicating that the protein-DNA interface has some accessibility to the solvent; this is discussed further below.

On reaching the four-way junction the DNA must undergo a substantial local and global structural reorganisation from the antiparallel stacked X-structure of the protein-free junction to the conformation observed in the crystal structure of the junction bound to endonuclease I^26^. Although the helical arms remain in two coaxial pairs, there is a rotation of ~120° between the axes. The enzyme remains fixed at the junction for an extended period (tens of seconds), although occasionally we observe endonuclease I break away from the junction and resume diffusion along the duplex DNA. If the enzyme is active (i.e., there is no active site mutation) and Mg^2+^ ions are present then cleavage can occur on average ~10 s after binding to the junction. This is consistent with the cleavage rate measured in free solution under single-turnover conditions of *k*_obs_ = 0.2 s^−1^. Separation of the beads attached to the DNA termini requires bilateral cleavage of the junction. For the majority of junction-resolving enzymes cleavage of the subsequent strand is accelerated relative to the first, thus helping to ensure a productive resolution of the junction^7,8^.

Crystal structures of T7 endonuclease I free^40^ and bound to a DNA junction^26^ suggest a manner of binding to duplex DNA. The dimeric protein generates two channels in the protein of 34 Å in length that are nearly perpendicular to each other. A four-way DNA junction binds such that pairs of coaxially-aligned helical arms lie in each channel of the protein (Fig. 6B). In our working model for the interaction of duplex DNA with endonuclease I, the dsDNA is bound in one channel of the dimeric protein and translocates along the DNA within that channel (Fig. 6B). There is significant local opening of the DNA structure when the junction is bound to endonuclease I (Fig. 6D). The opening is adjacent to the active site and is probably required for cleavage; when a simple duplex is accommodated into the channel the unperturbed B-DNA structure may prevent cleavage, explaining why we do not observe cleavage by active endonuclease I diffusing along duplex DNA. This ensures that only junctions are cleaved and ectopic cleavage of dsDNA is prevented. In the junction complex the protein is located on the minor groove side of the junction and surrounds each DNA arm on one side. While the bulk of the protein lies on the minor groove side of the junction, the major groove side of the junction probably interacts with the predominantly basic N-terminal peptides of 16 amino acids that are not observed in the crystal structures, likely due to dynamic or static disorder. It is known that the final C-terminal amino acid of the peptide joins to the N-terminus of the first alpha helix which is at the end of the electropositive clamp encircling the duplex arm. This places it immediately next to the opened major groove of the junction, and by far the most likely place for the peptide to bind would be in that major groove. Biochemical experiments have shown that the N-terminal tails are very important for binding to junctions^39^, and our single-molecule experiments confirmed their importance for binding with dsDNA. It is probable that if a duplex DNA binds to one of the protein channels then the N-terminal tail of the local domain wraps around the DNA, essentially encircling it (Fig. 6D). We have shown here that deletion of the N-terminal 16 amino acids leads to an impaired mobility on the DNA and a strong tendency to dissociate from the duplex DNA. Clearly, the N-terminal tail plays an important role in the stable binding to dsDNA and the lateral mobility of the enzyme.

We would expect the N-terminal peptide to continue the path of the electropositive helical clamp to encircle the DNA (probably in the major groove of the dsDNA), thereby closing off the DNA binding channel and preventing the protein from dissociation from the DNA. Encirclement of the open side of the DNA-binding channel would explain why the full-length enzyme can diffuse for multiple minutes on a long dsDNA yet is easily lost from short dsDNA molecules.

Calculation of the electrostatic potential of the surface of endonuclease I shows that it is divided into areas of very different overall charge characteristics (Fig. 6C). The floor of the binding channel is strongly electronegative, arising mainly from the active centre of the enzyme. This comprises a cluster of acidic amino acid side chains that coordinate the two catalytic metal ions. By contrast the ends of the channel are strongly electropositive. The orientation of the channel can be defined by the polarity of the continuous strand of the junction (see Fig. 6D). The end of the channel with the 3’ terminus has a series of basic side chains that include K67, R98, K100 and K123 that take a helical trajectory that follows the geometry of the DNA arm, forming an electropositive ‘clamp’ on the DNA. The lower end of this basic band ends with R16* (the asterisk * denotes the second polypeptide of the domain-exchanged dimer); this is the beginning of the basic N-terminal peptide that extends from this point (Fig. 6B). The other end of the channel (containing the 5’ end of the continuous strand of DNA) is also basic, with K40*, K145 and K103*. Thus, a DNA duplex bound to the enzyme will be held by electrostatic interactions at either end of the channel, and probably encircled by the N-terminal tail. It is possible that the DNA ‘floats’ in the varying electrostatic field of the channel, allowing the enzyme to undergo facilitated diffusion along the contour of the DNA. It is likely that this DNA-protein interface is accessible to metal ions, explaining why the measured rate of diffusion exhibits some dependence on the ionic strength of the medium. Indeed, we have previously noted that a complex of endonuclease I with a DNA junction formed in the absence of divalent metal ions can be activated by the addition of metal ions, indicating that they have access to the active site in the floor of the binding channel despite the presence of the DNA. We may perhaps draw an analogy with the diffusion of proliferating cell nuclear antigen (PCNA) along dsDNA; a toroidal homo-trimeric processivity factor that encircles dsDNA^44,45^ with the DNA located on its axis. PCNA undergoes 1D diffusion along DNA, the rate of which also exhibits some dependence on ionic strength^46^.

In summary, we have found that the phage junction-resolving enzyme locates a four-way junction by binding to dsDNA remotely from the junction and then tracking along the duplex by 1D diffusion until it encounters a junction. We have found a cellular resolving enzyme exhibits a similar 1D search, so it is likely this is general mechanism for junction-resolving enzymes. We have been able to observe a complete trajectory of resolution, from initial binding to duplex DNA, diffusion along the dsDNA, fixing to the four-way junction and ultimately cleavage of the DNA.

## Methods

### Oligonucleotides

Oligodeoxyribonucleotides were synthesised using standard phosphoramidite chemistry^47^ implemented on an Applied Biosystems 394 synthesiser and deprotected with ammonia at 55 °C for 2 h. All oligonucleotides were purified by electrophoresis in polyacrylamide gels containing 8 M urea. DNA was recovered from gel fragments by the crush and soak method followed by ethanol precipitation.

Modified phosphoramidites were purchased from Glen Research and are denoted with the following abbreviations: (5’-Biot) for 5’-Biotin phosphoramidite; (Biot-dT) for Biotin-dT phosphoramidite; (*ab*) for dSpacer CE phosphoramidite. **p** indicates 5’-phosphorylation.

Biot-F: (5’-Biot)-(Biot-dT)CA(Biot-dT)C(Biot-dT)GAAA CAGCAGCGGA

AS-R2: **p**CTTACAGATG (ab)CGCACGAAA AGCATCAGGT C

HX-J3-a: **p**ACATCTGTAA GAGTCTGCAG TTGAGTCCTT GCTAGGACGG ACGAAGTCCG TCCTAGCAAG GGGCTGCTAC CGGAAG

HX-J3-b: **p**ACATCTGTAA GCTTCCGGTA GCAGCCTGA GCGGTGGATG AACGAAGTTC ATCCACCGCT CAACTCAACT GCAGACT

### Construction of a DNA four-way junction for optical tweezers

A 7.5 kb handle was prepared by PCR using λ genomic DNA as a template and two modified primers, Biot-F and AS-R2 (above). Biot-F is fully complementary to the template and contains 4 biotin residues towards its 5’ end to enable bead attachment. AS-R2 is 5’-phosphorylated to enable ligation and contains an abasic site 10 nt from the 5’ end such that a proofreading polymerase will generate a 10 nt overhang^48,49^. We used Pfu Ultra II Fusion HS (Agilent) with the manufacturer’s recommended cycling parameters, and the PCR products were purified by application to a QIAquick PCR purification column (QIAGEN).

The phosphorylated, hairpin-forming, synthetic oligonucleotides HX-J3-a and HX-J3-b were annealed by slow-cooling an equimolar mixture from 80 °C down to room temperature in hybridisation buffer (10 mM Tris-HCl pH 7.5, 50 mM NaCl). This forms a four-way junction with one pair of opposite arms containing hairpins with a 15 bp stem capped by the 5 nt sequence CGAAG, known to form a very stable hairpin-turn structure^50^ while the other pair contains 15 bp and phosphorylated, 10 nt 5’-overhangs. These 5’-overhangs are identical and complementary to that of the handle to enable ligation.

The annealed junction was ligated to two handles by incubating 3 pmol of each with 400 u T4 DNA ligase (New England Biolabs) in ligase buffer at 16 °C for 8 h. The reaction was stopped by addition of 20 mM EDTA, 0.017% SDS and incubation at 65 °C for 10 min. Finally, the ligated substrate was purified by electrophoresis in a 0.6% agarose, 1x TAE gel containing 0.5x SYBR Safe DNA gel stain (Invitrogen), followed by electroelution of the excised product band and ethanol precipitation (Supplementary Fig. 1B, lanes 1 and 3). This substrate is cleaved efficiently by endonuclease I WT (Supplementary Fig. 1B, lane 4).

### Expression and labelling of T7 endonuclease I

The variants of endonuclease I used in this study were expressed from the plasmid pET-TEV-endoI^51^ engineered to remove the endogenous cysteine (C113S mutation) and add a new one at an accessible position remote from the DNA interacting surface (S29C mutation) for labelling. These mutations were shown previously not to affect significantly the activity of the expressed protein^41^. For simplicity, we refer to this version of endonuclease I as wild type. The active site mutant carried the additional D55A mutation, while the Δ16 mutant had 16 residues truncated from the N-terminus^39^.

BL21 (DE3) pLysS cells, transformed with the relevant plasmid, were grown at 37 °C to an A_600_ of 0.6 in LB medium supplemented with carbenicillin and chloramphenicol. They were then cooled to 25 °C, supplemented with 0.1 mM IPTG to induce protein expression and grown at 25 °C for a further 4 h. Cells were harvested by centrifugation at 8000 g for 30 min, washed with PS buffer (50 mM NaH_2_PO_4_/Na_2_HPO_4_ (pH 8), 1 M NaCl) and stored at −80 °C.

Cells pellets were thawed on ice, resuspended in 5 vol (w/v) of lysis buffer (PS supplemented with 20 mM imidazole, 0.2% Triton X100, cOmplete (EDTA-free) protease inhibitor cocktail (Roche) and 1 mg/ml lysozyme) and incubated at 20 °C for 30 min. The lysate was sonicated, clarified by centrifugation at 20,000 g, 4 °C for 30 min and applied to a His-Trap FF column (Cytiva) equilibrated in PS + 20 mM imidazole. His-tagged endonuclease I was eluted with a linear gradient from 20 mM to 1 M imidazole in PS buffer and dialysed into 50 mM Tris–HCl (pH 8.0), 100 mM NaCl, 1 mM DTT. The N-terminal oligohistidine tag was cleaved off by addition of His-tagged TEV protease to a molar ratio of 1:50 and incubation at 16 °C for 16 h. Finally, endonuclease I was purified on a His-Trap FF column as described above, dialysed into endonuclease I storage buffer (50 mM Tris-HCl pH 7.5, 40 mM NaCl, 30% glycerol) and stored at −80 °C.

Prior to labelling, endonuclease I was thawed on ice and incubated with 20 mM DTT on ice for 1 h to reduce its cysteine residues fully. The DTT was then removed by exchanging the buffer to labelling buffer (50 mM NaH_2_PO_4_/Na_2_HPO_4_, pH 7.2, 100 mM NaCl) using a HiTrap Desalting column (Cytiva). This was used to resuspend a 3- to 5-fold molar excess of Cy3 (or Cy5) maleimide (GE Healthcare) and the reaction mix was incubated at 20 °C for 16 h. The reaction was quenched by the addition of 1 mM DTT and the majority of the unincorporated dye was removed by buffer exchange into 20 mM NaH_2_PO_4_/Na_2_HPO_4_ (pH 6.0), 40 mM NaCl. The labelled protein was further purified on a HiTrap SP HP column (Cytiva) and eluted with a linear 40 mM – 2 M NaCl gradient in the same buffer. Finally, the protein was dialysed into endonuclease I storage buffer and stored at −80 °C. The purity of the endonuclease I variants used in this study is shown in (Supplementary Fig. 2A–C).

### Expression and labelling of CtGEN1

The expression plasmid pET-GEN1^28^ was modified by PCR to incorporate a ybbR tag^52^ in between residue 487 of C. thermophilum GEN1 and the C-terminal 6-histidine tag. The sequence beyond this residue reads GTDSLEFIASKLAGSHHHHHH, where the first serine is targeted by the labelling reaction.

Rosetta (DE3) cells (Novagen) transformed with this plasmid were grown at 37 °C to an A_600_ of 1.0 in LB medium supplemented with kanamycin and chloramphenicol. The temperature was then lowered to 25 °C and IPTG added to 0.25 mM to induce protein expression and the cells were cultured for a further 20 h under these conditions. Cells were harvested by centrifugation at 8000 g for 30 min, washed with Talon buffer (50 mM NaH_2_PO_4_/Na_2_HPO_4_, pH 8, 1 M NaCl, 10 mM imidazole) and stored at −80 °C.

Cells pellets were thawed on ice, resuspended in 5 vol (w/v) of lysis buffer (Talon buffer supplemented with 0.2% Triton X100, cOmplete (EDTA-free) protease inhibitor cocktail (Roche) and 1 mg/ml lysozyme) and incubated at 20 °C for 30 min. The lysate was sonicated, clarified by centrifugation at 20,000 g, 4 °C for 30 min and applied to a HiTrap TALON crude column (Cytiva) equilibrated in Talon buffer. CtGEN1 was eluted with a linear 10 mM to 1 M imidazole gradient and dialysed into 50 mM Hepes (pH 7.5), 100 mM NaCl for the labelling reaction.

Purified ybbR-tagged CtGEN1 was labelled enzymatically using the phosphopantetheinyl transferase Sfp and a Cy3-CoA conjugate^52^. His_6_-tagged Sfp was expressed from the plasmid pET29-Sfp^53^ in BL21 (DE3) cells, purified by affinity chromatography on a HisTrap FF column (Cytiva) and dialysed into 10 mM Tris-HCl (pH 7.5), 1 mM EDTA, 10% glycerol for storage at −80 °C. The Cy3-CoA conjugate was prepared by incubating 50 µg Cy3 maleimide (GE Healthcare) with 100 µg CoA sodium salt (Sigma) in 100 mM NaH_2_PO_4_/Na_2_HPO_4_ pH 7.1, 25% DMSO at room temperature for 1 h. The reaction product was purified by HPLC on a reversed phase C18 column (ACE) with a 5–50% acetonitrile gradient in 0.1 M TEAA and dried under vacuum. 1.5 µM CtGEN1 was incubated with 2 µM Cy3-CoA and 0.25 µM Sfp in 50 mM Hepes (pH 7.5), 100 mM NaCl, 10 mM MgCl_2_ at room temperature for 1 h, then purified by cation exchange chromatography on a HiTrap SP HP column (Cytiva). Finally, the labelled protein was dialysed into 20 mM Tris-HCl (pH 8.0), 200 mM NaCl, 10% glycerol for storage at −80 °C. The purity of Cy3-labelled CtGEN1 is shown in Supplementary Fig. 2D.

### Optical tweezers experiments

Single-molecule experiments were performed using a C-Trap (Lumicks) with integrated confocal microscopy and microfluidics, as described^20,30,31^. Prior to the experiment, all buffers were filtered with 0.2 μm syringe filters and microfluidic channels in the flow chamber (Fig. 1C) were passivated with Pluronics F128 (0.5% w/v in PBS) and BSA (0.1% w/v in PBS). Substrates were then introduced by flow into appropriate channels, as shown in Fig. 1C. Channel 1: 0.005% w/v streptavidin-coated bead particles in PBS (4.5 μm, Spherotech, cat no SVP-40-5). Channel 2: ~2 pM of DNA template with biotinylated ends (15 kb custom-made DNA with a four-way junction or 48.5 kbp λ-DNA, Life Technologies). Channel 3: 25 mM Tris-HCl (pH 7.5), 50 mM NaCl, 0.1% w/v BSA, 1 mM CaCl_2_. Channel 4: the same buffer as in Channel 3, supplemented with the protein of interest: 1 nM of WT-endonuclease I or 1 nM of endonuclease I-D55A or 1 nM Δ16-endonuclease I or 10 nM GEN1. A higher concentration of GEN1 was used due to its weaker binding affinity and the smaller labelled fraction of this sample. During cleavage experiments (Figs. 2A, 4C, Supplementary Fig. 5), 1 mM MgCl_2_ was included in the buffer instead of 1 mM CaCl_2_ to facilitate the enzymatic activity of WT-endonuclease I.

After optically trapping two beads (trap stiffness of 0.2–0.3 pN/nm) in Channel 1, a DNA molecule was tethered between the beads in Channel 2. The presence of the DNA tether was verified by measuring a force-extension curve in Channel 3 using constant pulling rate of 0.2 μm/s and the acquisition rate of 60 Hz (Fig. 1D). Subsequently, the DNA tether was moved to the intersection of Channel 3 and the protein channel (Channel 4) and the image acquisition was started. This allowed to capture the individual binding events of single proteins preceding their 1D diffusion and minimised background fluorescence. Both force-distance curves and images were recorded in the absence of flow in the microfluidic channel. Experiments with the WT endonuclease I were performed in a force-clamp mode with an active feedback loop to maintain constant force between the bead throughout imaging.

Confocal images were obtained by exciting the area of interest with a 532 nm or a 638 nm laser (Cy3 and Cy5 excitation, respectively, laser power < 3 μW). Fluorescence emission was detected in two channels with green (585/75 nm) and red (640LP) filters. Kymographs were acquired by scanning the DNA contour with a pixel dwell time of 0.1 or 0.2 ms/px (shorter dwell time for imaging λ-DNA) resulting in frame rates in the range of 40–100 ms.

### Data visualisation and analysis

Both force spectroscopy and fluorescence microscopy data were exported from LUMICKS Bluelake as.h5 files and subsequently processed with custom-written Python (Jupyter Notebook) scripts using Pylake package together with standard Numpy, Matplotlib, SciPy, PeakUtils libraries (https://github.com/singlemoleculegroup). Final plots were generated in Igor Pro 8 (Wavemetrics) and selected kymographs were cropped in FIJI.

Force-extension curves were plotted and analyzed with the extensible Worm-Like-Chain (eWLC) model^32^ to calculate the persistence length (*Lp* ~ 45 nm), contour length (*Lc*_*1*_ ~ 4.9 µm), and the stretch modulus (*S* ~ 1200 pN) of an optically suspended DNA molecule. The parameters were obtained by fitting the data points within the force range of 3–18 pN. To measure the distance change resulting from the unfolding of a four-way junction, the new contour length *Lc*_*2*_ was measured by fitting the eWLC model over the force range of 23–29 pN, using the *Lp* and *S* as fixed parameters obtained previously. The difference between the contour lengths (*Lp*_*2*_ *- Lp*_*1*_) indicates the size of the two unfolded DNA hairpins.

Confocal image and the corresponding force measurements were imported from an .h5 file and processed entirely in a single Jupyter Notebook. To evaluate endonuclease I docking on a four-way junction, a fragment of a kymograph with a static protein was integrated along the horizontal axis and cumulative fluorescence intensity was plotted. Pixel position was translated to genomic position (in kbp) using the centre of the beads (seen as blue peaks in the kymographs) as a reference point. The longest kymographs were integrated over 3–5 timeframes (down-sampled) to adjust the aspect ratio and the signal-to-noise ratio. Force measurement corresponding with each kymograph was down-sampled to 3 Hz.

### Single-particle tracking and diffusion analysis

To extract the position and the Mean Square Displacement (MSD) of endonuclease I, a custom-made single-particle tracking algorithm was incorporated within each Jupiter Notebook workspace. The kymograph imported from an .h5 file was cropped to eliminate the oversaturated parts of the image (i.e., the bead contours). The sub-pixel position of the fluorescent particle in every timeframe was calculated by fitting the signal intensity of a 3-timeframe moving window with 1D Gaussian function. The MSD analysis was performed on the trajectories that were longer than 10 s and did not cross with other molecules.

For every trajectory obtained, MSD was calculated as:

1 $${MSD}\, \left(n,N\right)=\mathop{\sum }\limits_{i=1}^{N-n}\frac{{({X}_{i+n}-{X}_{i})}^{2}}{N-n}=D{{{{{\rm{\tau }}}}}}\,+{{{{{\rm{b}}}}}},$$

where *N* is the total number of timeframes in the kymograph, *n* is the number of frames within a moving window (τ) from which the square displacement was calculated (ranging from 1 to *N*−1). *X*_*i*_ is the molecule position along the DNA at time *iI*, and b is the offset which is a measure for the accuracy. The diffusion coefficient of each individual molecule was obtained from the linear fit of the slope of the MSD curve (Fig. 3B). The fitting window was limited to 0.15 < τ < 1 s (3–10 lag times), to exclude errors emerging from stochastic variations (at the shortest lag times) and the confinement of the diffusion due to the limited length of the DNA (τ > 2 s). Gaussian tracking and MSD analysis of the static molecules docked to a four-way junction resulted in a frame-to-frame displacement of 30 nm (Supplementary Fig. 10) which is our localisation precision. The diffusion coefficients of a static molecule do not exceed D = 0.001 μm^2^/s = 0.008 · 10^6^ bp^2^·s^−1^ (20 times less than an average diffusion coefficient of a sliding endonuclease). From all translocating traces, we analysed the MSD dependency provided the standard error of the fit is < 0.001.

To estimate the speed of the protein, we measured the cumulative displacement of the trajectory that was smoothed with Savitzky–Golay filter and divided it over the time.

### Reporting summary

Further information on research design is available in the Nature Research Reporting Summary linked to this article.

## Supplementary information

Supplementary Information

Reporting Summary

## Data Availability

The raw data presented in the manuscript as force-distance curves, 2D confocal scans and kymographs have been deposited in the Zenodo database under accession code 7038534. Each datafile (.h5 file) can be accessed and processed by the corresponding Jupyter Notebook which is also available in the repository. Source data underlying the reported averages in Figs. 1E, 3E, F, 4D, E are provided as Source Data file. All remaining datasets are available upon request. Source data are provided with this paper.

## Source data

Source Data

## Code availability

The custom-made Python scripts written in Jupyter Notebook are available at www.github.com/singlemoleculegroup/Kaczmarczyk-et-al.-Search-and-Processing-of-Holliday-Junctions- and in the Zenodo repository.
