# Supplementary material for: Search and processing of Holliday junctions within long DNA by junction-resolving enzymes
Source: Nat Commun. 2022 Oct 7;13:5921. doi: 10.1038/s41467-022-33503-6 (PMC9547003; doi:10.1038/s41467-022-33503-6)
Supplement: Supplementary file 1 — Supplementary Information [file 41467_2022_33503_MOESM1_ESM.pdf]

Supplementary Information for

Search and Processing of Holliday Junctions within Long DNA by Junction-Resolving Enzymes

Artur P Kaczmarczyk[1,2], Anne-Cécile Déclais[3], Matthew D Newton[1,2,4], Simon J Boulton[4], David MJ Lilley[3,*] and David S Rueda[1,2,*]

[1] Department of Infectious Disease, Faculty of Medicine, Imperial College London, London W12 0NN, UK
[2] Single Molecule Imaging Group, MRC-London Institute of Medical Sciences, London W12 0NN, UK
[3] School of Life Sciences, University of Dundee, Dundee DD1 5EH, UK
[4] DSB Repair Metabolism Laboratory, The Francis Crick Institute, London, UK

* To whom correspondence should be addressed: d.m.j.lilley@dundee.ac.uk or david.rueda@imperial.ac.uk

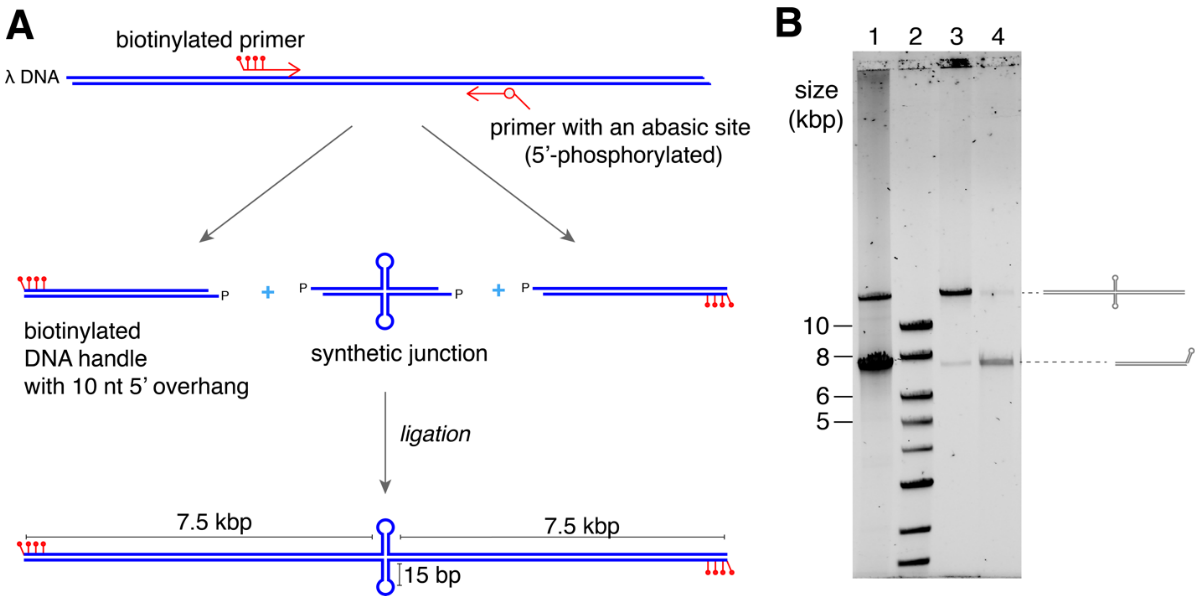

Supplementary Figure 1

Preparation of 15 kb dsDNA containing a four-way junction at its center. A. λ-DNA is used as a template to prepare 7.5 kb dsDNA handle. The fragment of interest is amplified via PCR reaction using one biotinylated primer and another 5'-phoshorylated primer containing an abasic site. Subsequently the PCR product is ligated with a double DNA hairpin that is formed by annealing two synthetic oligonucleotides. **B.** Resolution of the 15 kb four-way junction by Cy3-labelled endonuclease **I.** The gel-purified substrate (lane 3) was digested at room temperature for 30 s with unlabelled endonuclease I (lane 4). The 15 kb substrate is cleaved to 79% by the labelled enzyme and to 89% by the unlabelled protein. Reaction products were run on a 0.6% agarose gel containing the SYBR Safe stain and visualised with a fluorimager using a 473 nm laser and a 510LP filter. Appearance of a 7.5 kb product band indicates bilateral cleavage of the central four-way junction. Lane 1: ligation reaction; lane 2: molecular weight marker (1 kb DNA ladder, New England Biolabs).

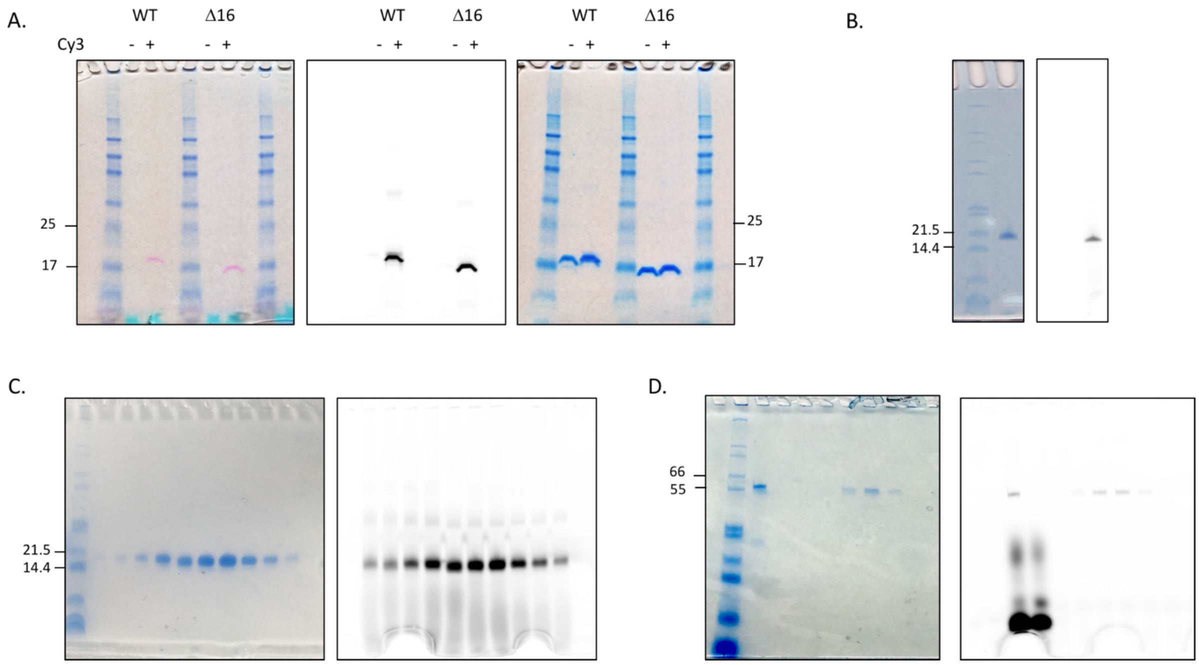

Supplementary Figure 2

Purity of the proteins used in this study. Each panel shows the same gel imaged in different conditions. Fluorescence scans were performed on the unstained gels with a Typhoon FLA 9500 fluorimager using a 532 nm laser and a 570DF20 filter for Cy3 and a 635 nm laser and a 665LP filter for Cy5. The molecular weight (kDa) of relevant marker bands is indicated next to the gel. **A.** Cy3-labelled endonuclease I WT and Δ16. Unlabelled (-) and Cy3-labelled (+) proteins are run side-by-side in between ladders of prestained molecular weight markers. Left panel: unstained gel, showing the Cy3-labelled proteins as pink bands. Middle panel: Cy3 fluorescence scan. Right panel: Coomassie stained gel. Labelling fractions: Cy3-WT, 72%, Cy3-Δ16, 56%. **B.** Cy3-labelled endonuclease I d55A. Left panel: Coomassie stained gel. Right panel: Cy3 fluorescence scan. **C)** Cy5-labelled endonuclease I d55A. The gel shows fractions eluted from a cation exchange column following the labelling reaction. Left panel: Coomassie stained gel. Right panel: Cy5 fluorescence scan. Labelling fractions: Cy3-D55A, 80%, Cy5-D55A, 88% **D)** Cy3-labelled CtGEN1. The gel shows the purification of the labelled protein on a cation exchange column. Lane 1, labelling reaction; lane 2, column flow-through; following lanes, eluted fractions. Left panel: Coomassie stained gel. Right panel: Cy3 fluorescence scan. Labelling fraction Cy3-GEN1, 10%.

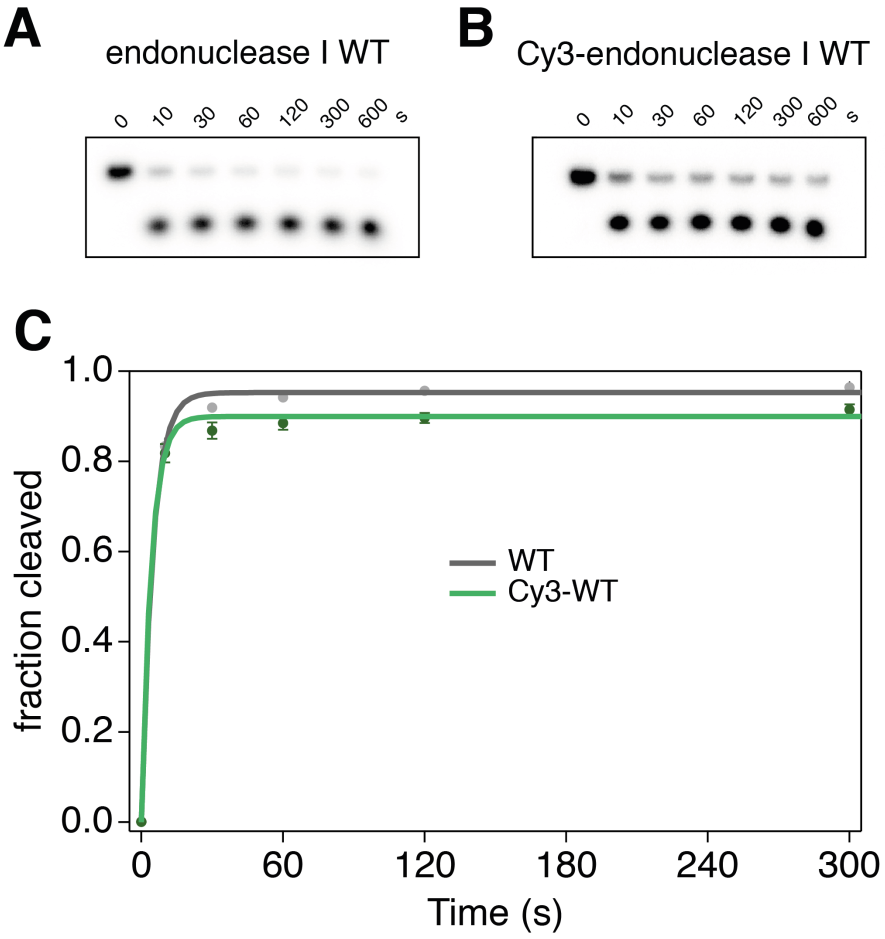

Supplementary Figure 3

Comparison of the catalytic activity of unlabelled and Cy3-labelled endonuclease I WT. A four-way junction comprised of 15 bp arms was radioactively 5'-^{32}P-labelled on a single strand (x-strand) and cleaved in single turnover conditions by either protein on ice and in the presence of magnesium ions. The reaction products were resolved by electrophoresis in denaturing 15% polyacrylamide gels and analysed by phosphorimaging. Each experiment was performed in triplicates. **A.** and **B.** Phosphorimage of representative gels for unlabelled endonuclease I and Cy3-labelled endonuclease I, respectively. The lower bands represent the reaction product. Time points, 0, 10 s, 30 s, 1 min, 2 min, 5 min and 10 min. **C.** The time course of the reaction is plotted as fraction cleaved versus time (mean of triplicates). Black circles, endonuclease I WT. Green circles, Cy3-labelled endonuclease I WT. The error bars represent standard deviations and the line are best fits to single exponentials. The rate constants obtained were 0.21 s^{-1} for endonuclease I WT and 0.24 s^{-1} for Cy3-labelled endonuclease I WT.

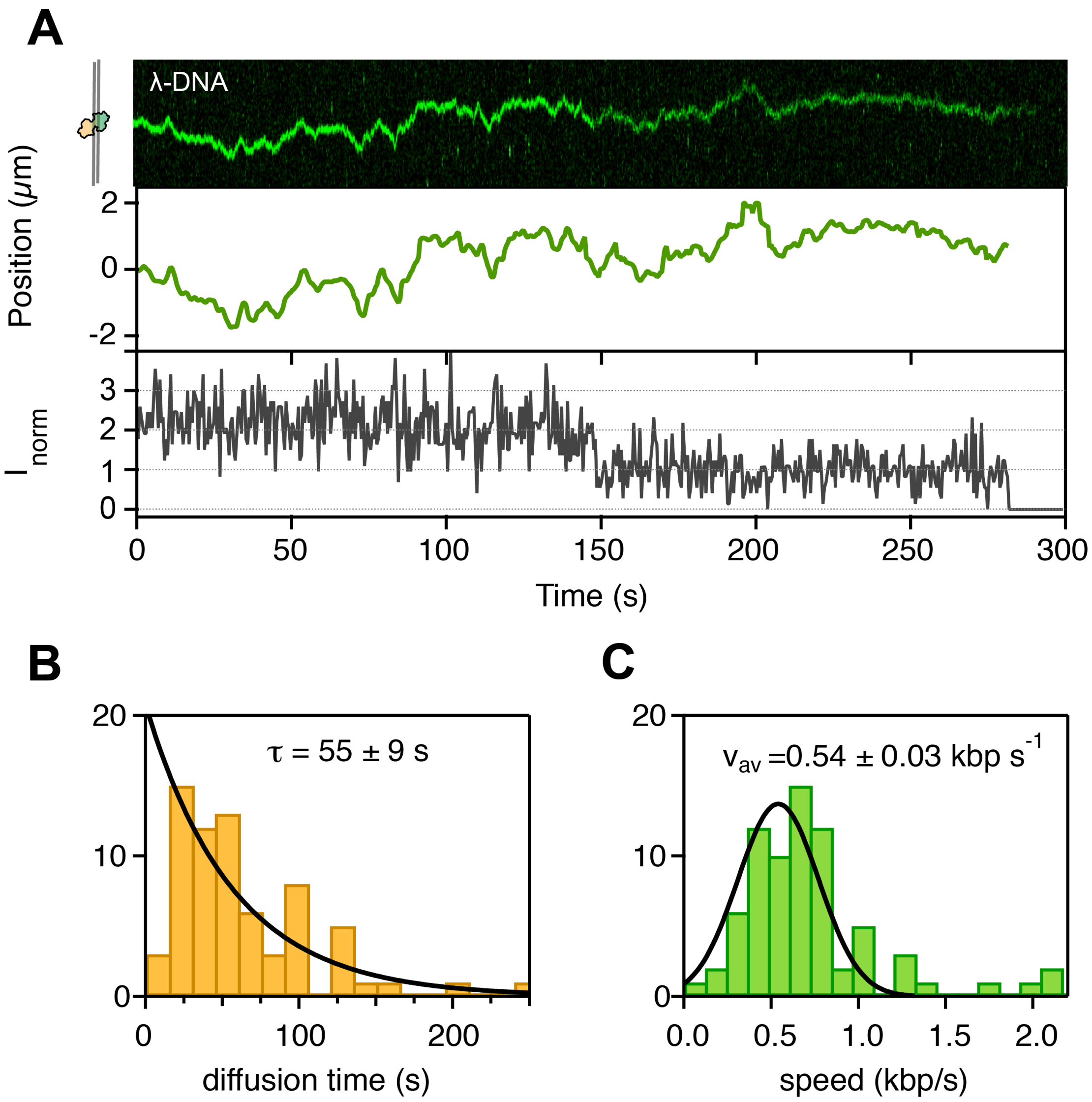

Supplementary Figure 4

Quantitative analysis of endonuclease I complexes undergoing 1D diffusion.

A. A kymograph showing a two-step photobleaching of the Cy3-labeled endonuclease I dimer diffusing o a λ-DNA (a template lacking four-way junctions). *Middle panel*: Trajectory of the molecule resolved by single-particle Gaussian tracking. *Bottom panel*: Normalized intensity of the pixels detected by the Gaussian tracking. Intensity drops at 150th and 280th second correspond to photobleaching events of the two Cy3 dyes conjugated with the endonuclease I homodimer. **B.** Distribution of endonuclease I diffusion times in 50 mM NaCl buffer, and the exponential fit (black line) yielding the half time τ = 55 ± 9 s (n = 69). Right panel: distribution of endonuclease I velocities at 50 mM NaCl and Gaussian fit (black line) yielding mean speed of 0.54 ± 0.03 kbp/s.

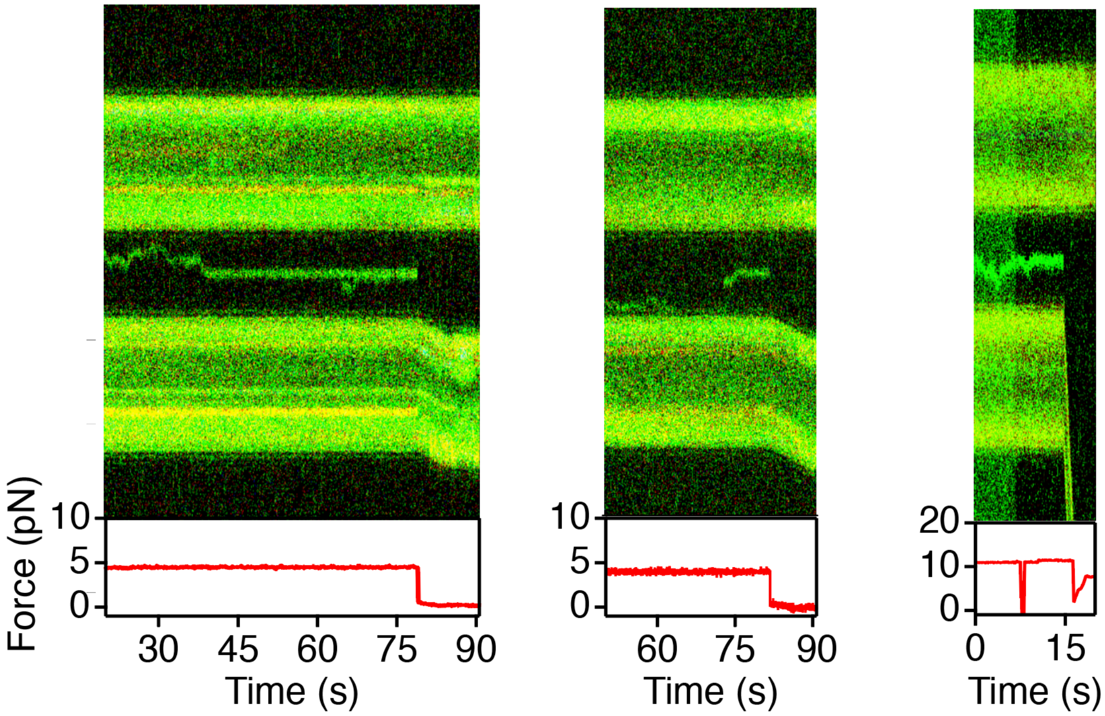

Supplementary Figure 5

Complete reaction trajectories of endonuclease I dimers observed on individual DNA tethers. Kymographs above show single endonuclease I dimers that diffuse on the dsDNA until encountering a four-way junction which leads to the cleave of the DNA. Corresponding force read-out in force clamp mode is plotted below each kymograph. Spike in the force in the right panel is due to the movement of the tether within the microfluidic channel.

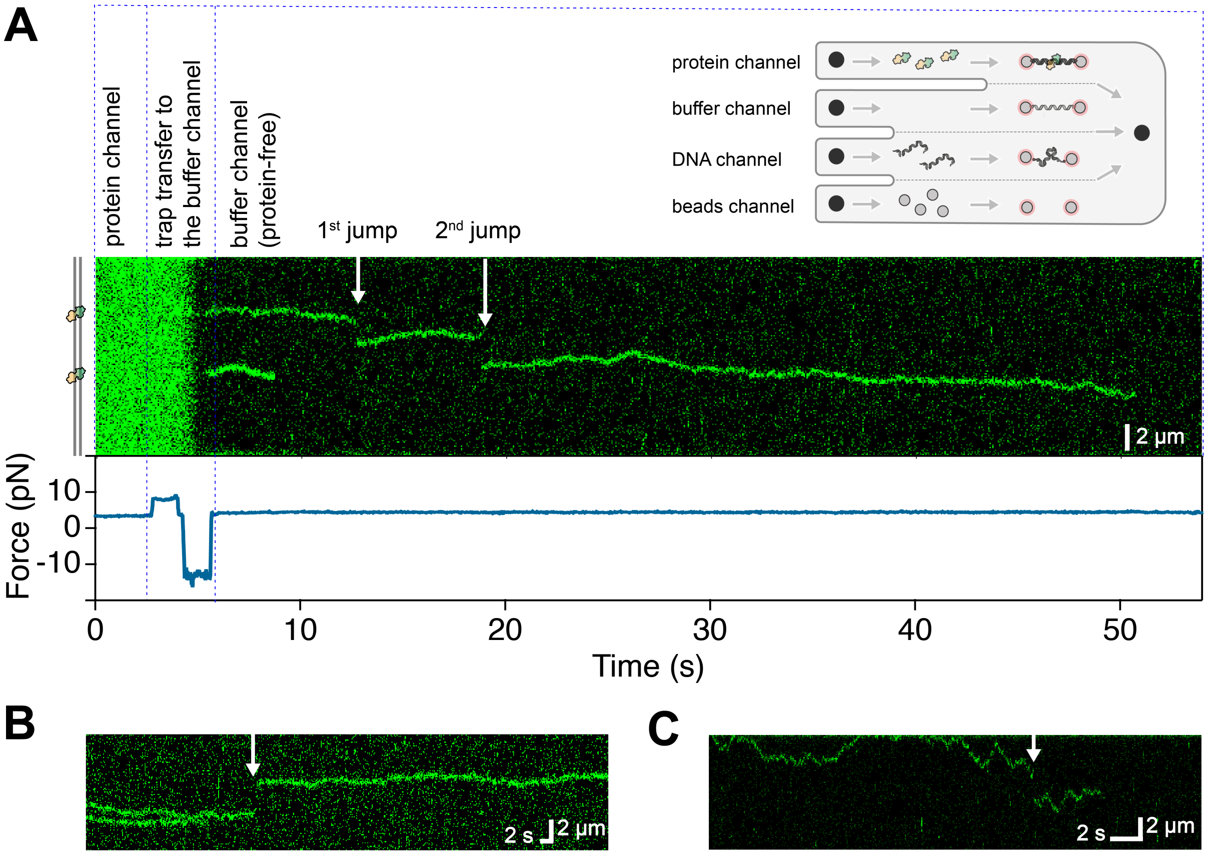

Supplementary Figure 6

Endonuclease I performs long hops of 1-3 µm. A. A kymograph and the corresponding force measurement of endonuclease I diffusing on λ-DNA. Initially, the kymograph was recorded in the protein channel, and subsequently moved to the imaging channel. Throughout the experiment, the DNA was under 5 pN force, except the moment when the tether was moved between channels.

In 13th and 18th second, the diffusing endonuclease moves to another segment of the DNA within a single time frame (100 ms per frame). **B.** and **C.** kymographs showing different examples of intersegmental hops of endonuclease I.

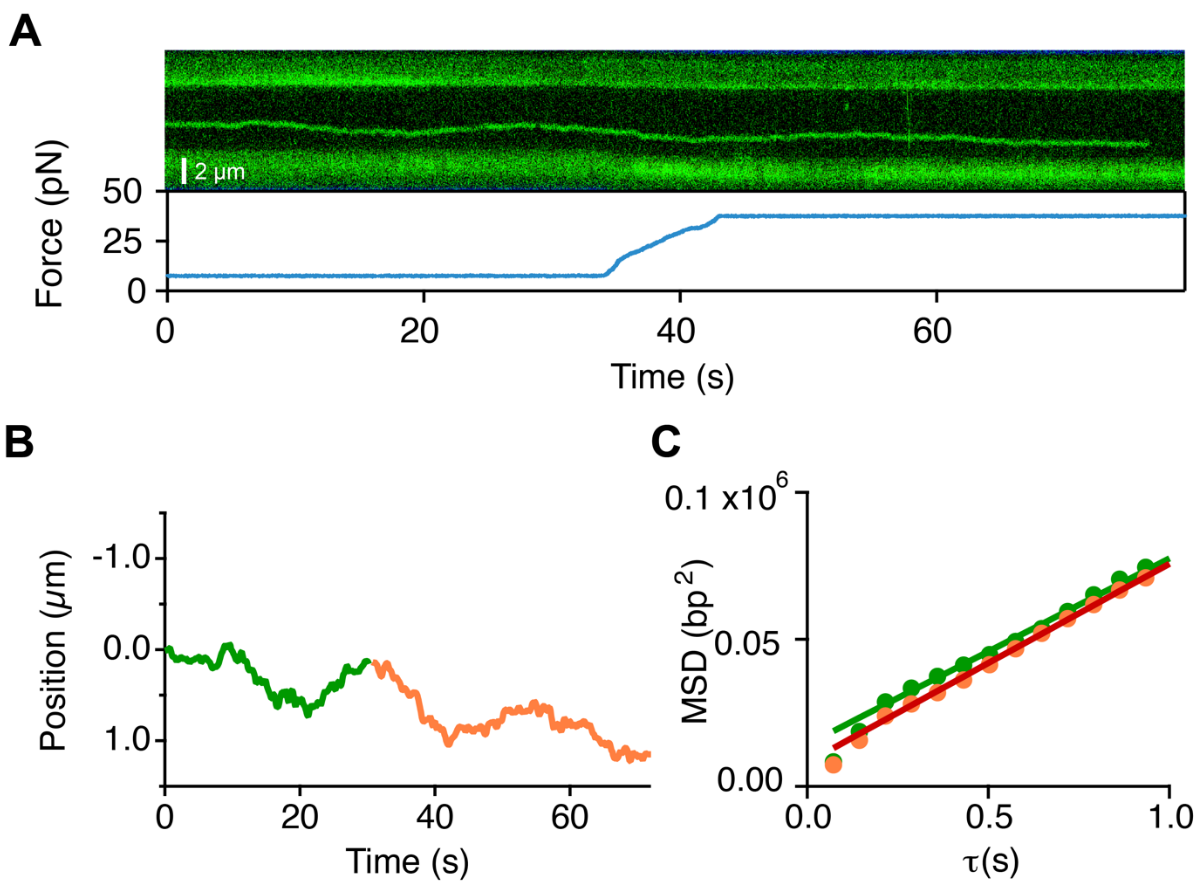

Supplementary Figure 7

Diffusion coefficient does not depend on force applied on the DNA tether. A. A kymograph and the corresponding force read-out of endonuclease I diffusing on a dsDNA. After 40 seconds of acquisition at 5 pN, the tethered DNA was stretched to 40 pN. **B.** The trajectory shown in panel A resolved by single-particle Gaussian tracking separated into two parts: diffusion at low force (green) and diffusion at high force (orange). **C.** Mean-square displacement (MSD) plot of the trajectories shown in panel B. Linear fit to the MSD dependency yields the diffusion coefficient of endonuclease I (green, low force: $D = 0.0037 \pm 0.001\ \mu m^2/s = 0.3 \cdot 10^5\ bp^2/s$, orange: $D = 0.0040 \pm 0.001\ \mu m^2/s = 0.3 \cdot 10^5\ bp^2/s$).

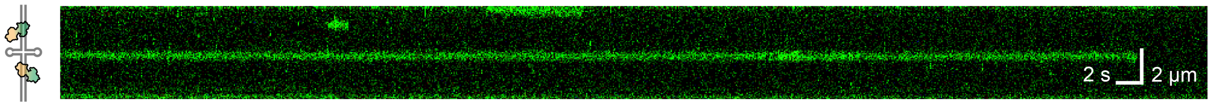

Supplementary Figure 8

Δ16 endonuclease I docks stably on a four-way junction. A kymograph showing Δ16 endonuclease bound to the center of the DNA tether. Only two short binding events occurred on the duplex DNA.

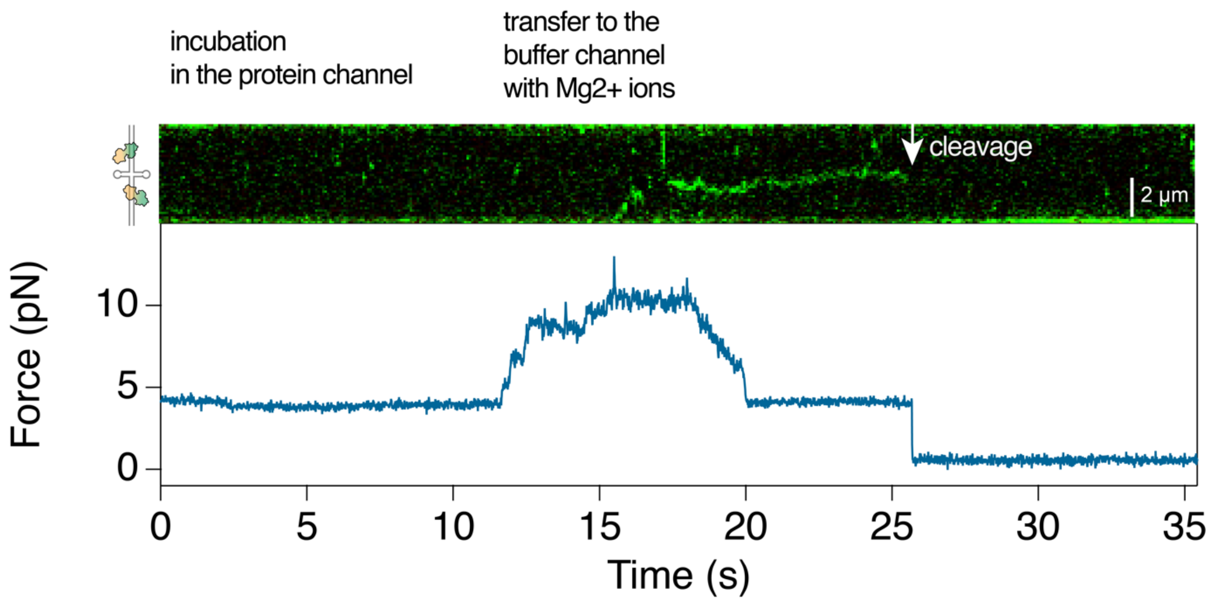

Supplementary Figure 9

GEN1 diffuses on the duplex DNA and executes nucleolytic cleavage once it docks on a four-way junction. A kymograph showing GEN1 molecule diffusing on the DNA with a four-way junction. The protein binds to the duplex DNA in the 16th second of imaging. Once it reaches the center of the tether, the fluorescent signal disappears, indicating the cleavage of dsDNA. This coincides with the force dropping to 0 pN, as shown in Figure 2 and 4C. The change in force between 12-20th second results from the movement of the optical trap from the protein channel to the buffer channel.

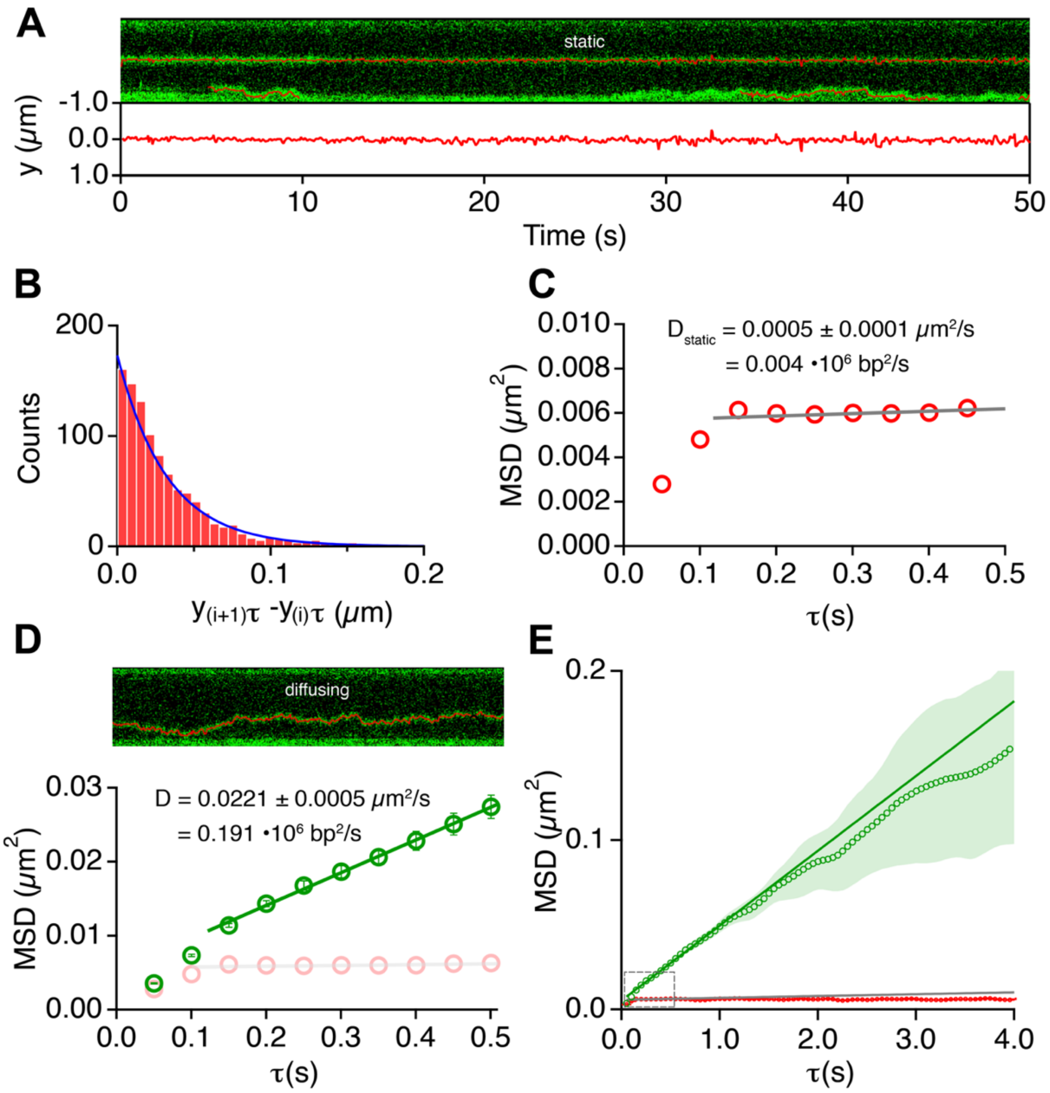

Supplementary Figure 10

Single-particle Gaussian tracking algorithm enables quantification of mean-square displacement (MSD) with sub-pixel resolution. A. *Top*: A kymograph with trajectories recognized by the algorithm (drawn as red traces). *Bottom*: The trajectory of the static endonuclease I extracted from the kymograph above. **B.** Distribution of endonuclease I displacements per single timeframe frame of the kymograph shown in panel A. The blue line represents the exponential fit. The image was recorded with pixel size = 0.05 µm/px. **C)** Mean-square displacement (MSD) plot of the trajectory shown in panel A. Linear fit to the MSD dependency over intervals 0.15 s $\leq \tau \leq$ 0.5 s yields the apparent diffusion coefficient of the static endonuclease I (D = 0.0005 ± 0.0001 $\mu m^2 s^{-1}$, (S.E.)). **D)** Mean-square displacement (MSD) plot of the trajectory shown in the inset kymograph. Linear fit to the MSD dependency over intervals 0.15 s $\leq \tau \leq$ 0.5 s yields the diffusion coefficient of the mobile endonuclease I (D = 0.0221 ± 0.0005 $\mu m^2 s^{-1}$, (S.E.)). Error bars represent variance of the mean-square displacement at each τ point. **E)** Mean-square-displacement dependencies shown in panel C and D plotted over a wider range of the time intervals ($\tau \leq 4$ s). The grey dotted box indicates the range of the data chosen for the MSD analysis. The green shading represents the variance of MSD.
