# Supplementary material for: Search and processing of Holliday junctions within long DNA by junction-resolving enzymes
Source: Nat Commun. 2022 Oct 7;13:5921. doi: 10.1038/s41467-022-33503-6 (PMC9547003; doi:10.1038/s41467-022-33503-6)
Supplement: Supplementary file 2 — Reporting Summary [file 41467_2022_33503_MOESM2_ESM.pdf]

nature portfolio

Corresponding author(s): Lilley DMJ and Rueda DS

Last updated by author(s): Sep 2, 2022

Reporting Summary

Nature Portfolio wishes to improve the reproducibility of the work that we publish. This form provides structure for consistency and transparency in reporting. For further information on Nature Portfolio policies, see our Editorial Policies and the Editorial Policy Checklist.

Statistics

For all statistical analyses, confirm that the following items are present in the figure legend, table legend, main text, or Methods section.

n/a	Confirmed	
☐	☒	The exact sample size (*n*) for each experimental group/condition, given as a discrete number and unit of measurement
☐	☒	A statement on whether measurements were taken from distinct samples or whether the same sample was measured repeatedly
☐	☒	The statistical test(s) used AND whether they are one- or two-sided<br>*Only common tests should be described solely by name; describe more complex techniques in the Methods section.*
☐	☒	A description of all covariates tested
☒	☐	A description of any assumptions or corrections, such as tests of normality and adjustment for multiple comparisons
☐	☒	A full description of the statistical parameters including central tendency (e.g. means) or other basic estimates (e.g. regression coefficient) AND variation (e.g. standard deviation) or associated estimates of uncertainty (e.g. confidence intervals)
☐	☒	For null hypothesis testing, the test statistic (e.g. *F*, *t*, *r*) with confidence intervals, effect sizes, degrees of freedom and *P* value noted<br>*Give P values as exact values whenever suitable.*
☒	☐	For Bayesian analysis, information on the choice of priors and Markov chain Monte Carlo settings
☒	☐	For hierarchical and complex designs, identification of the appropriate level for tests and full reporting of outcomes
☒	☐	Estimates of effect sizes (e.g. Cohen's *d*, Pearson's *r*), indicating how they were calculated

Our web collection on statistics for biologists contains articles on many of the points above.

Software and code

Policy information about availability of computer code

Data collection	Single-molecule data was collected using the commercial software Bluelake v. 1.6 (LUMICKS) that is fully compatible with the instrument (correlative optical tweezer and confocal microscope C-TRAP). Measurements of individual DNA molecules were saved as .H5 Hierarchical Data Format. All H5-files contain force and position data of optically trapped beads, as well as pixel values of the recorded 3-color confocal images. The exported files contain additionally meta-data such as: experimental description, status of the microfluidic system, laser powers, laser coordinates, camera settings.
Data analysis	All data were processed and analyzed using custom-made scripts written in Python v. 3.9 using Pylake 0.10.1/Numpy 1.20.3/Matplotlib 3.4.2/ Scipy 1.6.3/Peakutils 1.3.3. packages.<br>Each H5-file was processed in a separate Jupyter Notebook - an interactive platform to visualize the results of the Python script. Within a single Jupyter Notebook, the fluorescent data was rendered into an RGB image, was correlated with the force measurement, subsequently underwent the single-particle tracking and mean-square-displacement (MSD) analysis. A representative Jupyter Notebook that includes all the above features is available at www.github.com/singlemoleculegroup.<br>Selected images rendered in Jupyter Notebook into .TIFF images were cropped in ImageJ 2.1<br>Datafiles that contained only force & position measurement (without fluorescent images, for example data in Figure 1C) were analyzed in a single Jupyter Notebook for batch analysis of many individual DNA molecules (www.github.com/singlemoleculegroup).<br>Commercial software Wavemetrics IGOR 8 was used to generate final plots (force-distance curves, 1D trajectories, MSD plots and histograms). To do so, the output of each Jupyter Notebook (in the .CSV format) was imported to IGOR's workspace.<br>Structures of Endonuclease I complexes were rendered with ChimeraX (Figure 1a) and in PyMol (Figure 6)<br>Final figures were made in Adobe Illustrator 2020.

For manuscripts utilizing custom algorithms or software that are central to the research but not yet described in published literature, software must be made available to editors and reviewers. We strongly encourage code deposition in a community repository (e.g. GitHub). See the Nature Portfolio guidelines for submitting code & software for further information.

Data

Policy information about availability of data

All manuscripts must include a data availability statement. This statement should provide the following information, where applicable:

- Accession codes, unique identifiers, or web links for publicly available datasets
- A description of any restrictions on data availability
- For clinical datasets or third party data, please ensure that the statement adheres to our policy

The raw data presented in the manuscript as force-distance curves, 2D confocal scans and kymographs have been deposited in the Zenodo database at https://doi.org/10.5281/zenodo.7038534
Source data underlying the reported averages in Figures 1E, 3 E-F, 4 D-E are provided as Source Data file. All remaining datasets are available upon request.

Field-specific reporting

Please select the one below that is the best fit for your research. If you are not sure, read the appropriate sections before making your selection.

☒ Life sciences ☐ Behavioural & social sciences ☐ Ecological, evolutionary & environmental sciences

For a reference copy of the document with all sections, see nature.com/documents/nr-reporting-summary-flat.pdf

Life sciences study design

All studies must disclose on these points even when the disclosure is negative.

Sample size	No sample size calculation was performed. All observations were repeated on sufficient numbers of individually captured DNA-protein complexes. It was determined by the number of trapped single-molecules of DNA in the combined dataset for each condition.<br>In our study, we captured single DNA molecules by an optical tweezer and probed the DNA interaction with multiple fluorescent protein molecules in real-time. We measured separately dozens of individual DNA tethers in each condition. About 40 individual molecules were measured independently to assess the unfolding of a Holliday Junction. Gaussian distribution of the obtained results, as well as proper Worm-Like-Chain model parameters characterizing the DNA convinced us that it is a sufficient sample size.<br>About 50-100 individual DNA molecules (either with or without Holliday Junction) were captured in each experimental condition for the purpose of the diffusion analysis. Having suspended a single DNA molecule, we could record trajectories of multiple proteins interacting with one DNA strand, giving us diffusion rates of ~100 single protein complexes. About ~40 DNA molecules were captured separately for the purpose of the cleavage experiments.
Data exclusions	1) Kymographs that contained too many diffusing species were excluded because the protein molecules were colliding with each other, obscuring the results of a single-particle tracking algorithm.<br>2) Kymographs recorded for the cleavage experiments were excluded if the endonuclease I was not visibly bound to the Holliday Juction prior to the DNA cleavage (most likely the protein bound to the target site was photobleached).<br>3) Trajectories of the protein diffusing on the DNA were not included in the statistics if the diffusion was shorter than 5 seconds (not enough datapoints to fit linear mean-square-displacement)
Replication	On each experimental day, we captured ~10-30 DNA molecules (one by one) that had a proper contour length (5.1 um and 16.5 um for Holliday Junction DNA and lambda DNA, respectively) and appropriate mechanical properties (persistence length, stretch modulus characteristic to dsDNA). Each experiment was replicated in at least 3 independent sessions. The resulting force-distance curves are practically identical, and are easily reproducible.<br>Each kymograph showing endonuclease I bound to DNA is an unique image representing a random walk of protein complexes on the DNA. The observed binding dwell times, diffusion rates are reproducible within the standard deviation.
Randomization	Randomization was not relevant in this study. We analyzed the DNA-protein interaction on each, individual molecule trapped in optical tweezers.
Blinding	Blinding was not relevant to this study. Experiments were performed on single molecules of DNA in contact with fluorescent proteins.

Reporting for specific materials, systems and methods

We require information from authors about some types of materials, experimental systems and methods used in many studies. Here, indicate whether each material, system or method listed is relevant to your study. If you are not sure if a list item applies to your research, read the appropriate section before selecting a response.

Materials & experimental systems

n/a	Involved in the study
☒	☐ Antibodies
☒	☐ Eukaryotic cell lines
☒	☐ Palaeontology and archaeology
☒	☐ Animals and other organisms
☒	☐ Human research participants
☒	☐ Clinical data
☒	☐ Dual use research of concern

Methods

n/a	Involved in the study
☒	☐ ChIP-seq
☒	☐ Flow cytometry
☒	☐ MRI-based neuroimaging
